# Comparative Efficacy and Safety of Ciprofol Versus Propofol in Adults Undergoing Fiberoptic Bronchoscopy: A Systematic Review and Meta-Analysis of Randomized Controlled Trials

**DOI:** 10.3390/ph19101575

**Published:** 2026-10-04

**Authors:** Sichen Sun, Rui Wang, Mengling Ouyang, Minghui Sun

**Affiliations:** 1 Department of Pharmacy, Tongji Hospital, Tongji Medical College, Huazhong University of Science and Technology, Wuhan 430000, China; sscSCMZU@163.com; 2 School of Pharmacy, Tongji Medical College, Huazhong University of Science and Technology, Wuhan 430000, China; u202213213@hust.edu.cn

**Keywords:** ciprofol, propofol, fiberoptic bronchoscopy, sedative efficacy, safety, meta-analysis

## Abstract

**Background:** Ciprofol, a novel intravenous sedative, has recently gained increasing clinical attention for procedural anesthesia. Emerging evidence suggests it may possess potential safety advantages over propofol. This study aimed to systematically compare the efficacy and safety of ciprofol and propofol for sedation in patients undergoing fiberoptic bronchoscopy. **Methods:** A comprehensive literature search was conducted in eight electronic databases. Statistical analyses were performed using the R software (version 4.5.2). The risk ratio (RR) with 95% confidence interval (CI) was used for dichotomous outcomes, and the mean difference (MD) with 95% CI was used for continuous outcomes. **Results:** This meta-analysis included 22 RCTs with 2452 participants. Compared with propofol, ciprofol significantly reduced the incidence of hypotension (RR, 0.39; 95% CI, 0.28–0.55), injection pain (RR, 0.14; 95% CI, 0.10–0.19), hypoxemia (RR, 0.56; 95% CI, 0.35–0.90), and respiratory depression (RR, 0.23; 95% CI, 0.12–0.43). No significant differences were found in cough (RR, 1.01; 95% CI, 0.89–1.14), bradycardia (RR, 0.69; 95% CI, 0.42–1.14), nausea and vomiting (RR, 0.84; 95% CI, 0.56–1.27), induction time (MD, 0.03; 95% CI, −10.57–10.62), operation time (MD, −0.3060; 95% CI, −0.6127–0.0008), recovery time (MD, 0.10; 95% CI, −0.59–0.79), or procedural success rate (RR, 1.00; 95% CI, 0.99–1.01). **Conclusions**: Ciprofol provides comparable sedative efficacy to propofol for fiberoptic bronchoscopy, while reducing the incidence of adverse events, suggesting it may be a favorable alternative.

## 1. Introduction

With the rapid development of interventional pulmonology, fiberoptic bronchoscopy plays a very important role in the diagnosis of various lung diseases, including lung cancer, sarcoidosis, and pulmonary tuberculosis [1,2,3,4]. However, as an invasive procedure, fiberoptic bronchoscopy can induce several adverse effects such as coughing, nausea, and vomiting. These complications may impede procedural progress and reduce patient comfort during examination [5]. Therefore, safe and effective sedation and anesthesia are crucial for ensuring both the success of the operation and patient tolerance and experience. Among the available sedatives, propofol is commonly used for fiberoptic bronchoscopy owing to its rapid onset, short duration of action, and rapid recovery profile [6,7,8]. Nevertheless, its use is limited by notable side effects, including respiratory depression, hypotension, and injection pain [9,10]. It appears that many patients undergoing fiberoptic bronchoscopy are middle-aged or elderly, and related anesthetic side effects may increase procedural risks [11,12]. Consequently, this scenario may bring challenges for clinicians, prompting the search for alternative therapeutic agents.

Ciprofol, a novel intravenous sedative, shares a similar chemical structure with propofol. Both agents primarily act by activating the effects of gamma-aminobutyric acid (GABA) receptors in the central nervous system, facilitating rapid induction and maintenance of sedation [13]. However, preclinical studies suggest that ciprofol demonstrates superior efficacy to propofol and induces fewer respiratory and circulatory depressive effects [14]. Owing to its higher receptor affinity, ciprofol has a lower effective dose than that of propofol. These pharmacological advantages were corroborated by clinical evidence. For instance, a meta-analysis by Yu et al. reported that ciprofol was associated with a significantly lower incidence of hypotension, respiratory depression, and injection pain than propofol [15]. Moreover, several other studies have indicated that ciprofol possesses a broader therapeutic index than propofol, conferring a more favorable safety profile in the context of procedural sedation [16,17].

Despite these promising pharmacological and clinical insights, evidence specifically pertaining to bronchoscopy remains limited. Although several systematic reviews have compared ciprofol and propofol in other settings such as gastrointestinal endoscopy and general anesthesia, there is currently no dedicated meta-analysis focusing exclusively on bronchoscopy [5,18]. Furthermore, the results of multiple randomized controlled trials (RCTs) regarding ciprofol remain inconsistent. Therefore, we conducted this meta-analysis to comprehensively compare the efficacy and safety of ciprofol versus propofol for sedation during fiberoptic bronchoscopy with the aim of providing reliable guidance for clinical practice.

## 2. Materials and Methods

### 2.1. Study Registration

This meta-analysis followed the guidelines of the Preferred Reporting Items for Systematic Reviews and Meta-Analyses (PRISMA) 2020 statement (Table S1) [19]. The study protocol was registered in the International Prospective Register of Systematic Reviews (PROSPERO) under the registration number CRD420251185702.

### 2.2. Literature Search

A comprehensive literature search was conducted in PubMed, Web of Science, Embase, Cochrane Library, Scopus, China National Knowledge Infrastructure (CNKI) database, Wanfang Database, and *ClinicalTrials.gov* (accessed on 1 December 2025), without language restrictions, from inception to December 2025. The search keywords included the following Medical Subject Headings (MeSH) and their synonyms: Ciprofol, fiberoptic bronchoscopy, and randomized controlled trials and their types. The detailed search information is presented in Table S2.

### 2.3. Inclusion and Exclusion Criteria

The inclusion criteria were as follows: (a) Population: Adult patients (≥18 years) undergoing fiberoptic bronchoscopy. (b) Intervention: Sedation using ciprofol (any dose or regimen). (c) Comparator: Sedation using propofol (any dose or regimen). (d) Outcomes: Primary outcomes included the incidence of intraprocedural hypotension as defined by each individual study; secondary outcomes comprised the incidence of injection pain, hypoxemia, coughing, bradycardia, nausea and vomiting, respiratory depression, induction time, operation time, recovery time, and procedural success rate. The included studies were required to report at least one pre-specified primary or secondary outcome. (e) Study design: Randomized controlled trials published as full-text articles in peer-reviewed journals.

The exclusion criteria were as follows: (a) Duplicated publications; (b) Non-RCT study designs, including reviews, retrospective studies, and other observational studies; (c) Unpublished or ongoing studies; and (d) Studies with incomplete or unavailable key data.

### 2.4. Literature Selection and Data Extraction

All the literature was imported into Zotero (version 7.0.30) for management. Two researchers first independently screened titles and abstracts, and then reviewed the full-text manuscripts based on the inclusion and exclusion criteria. Any discrepancies were resolved through discussion, and a third researcher was consulted if needed.

Two researchers independently retrieved all relevant information from the included studies using a pre-designed data extraction form specifically developed for this meta-analysis. The following data were extracted from each RCT: First author, publication year, participant characteristics (sample size, sex, age, and BMI), ASA classification, intervention details, comparator details, and outcome data. The number of events and the total number of patients assessed were extracted for dichotomous outcomes (e.g., incidence of hypotension and injection pain). For continuous outcomes (e.g., induction time and operation time), the mean, standard deviation (SDs), and sample sizes were collected. When continuous data were reported in the form of median, interquartile range (IQR), or range, the mean and SDs were estimated using the methods proposed by Wan et al. and Luo et al. [20,21].

### 2.5. Quality Assessment

Two researchers independently assessed the risk of bias of the included RCTs using Cochrane Risk of Bias (ROB) Tool 2.0 [22]. The assessment covered five key domains: (a) Randomization process; (b) Deviations from the intended interventions; (c) Missing outcome data; (d) Measurement of the outcomes; and (e) Selection of the reported results. The overall risk of bias was categorized into three levels: Low risk, some concerns, or high risk.

### 2.6. Certainty of the Evidence

The Grading of Recommendations, Assessment, Development, and Evaluations (GRADE) framework was applied to assess the certainty of evidence for all pooled outcomes [23]. Evidence from RCTs starts at a high-certainty baseline and may be downgraded for five domains: Risk of bias, inconsistency, indirectness, imprecision, and publication bias [24,25]. Downgrading considerations included suboptimal sequence generation, inadequate blinding, baseline imbalance, substantial statistical heterogeneity, wide confidence intervals, and failure to reach the optimal information size (OIS). We generated the evidence-certainty summary table using GRADEpro software (version 3.6). Evidence certainty was categorized as high, moderate, low, or very low.

### 2.7. Statistical Analysis

All statistical analyses were performed using R (version 4.5.2), R Studio (2025.09.2), and meta package. For dichotomous outcomes, the risk ratio (RR) with a 95% confidence interval (CI) was used as the effect measure. For continuous outcomes, the mean difference (MD) with a 95% CI was applied. Considering clinical heterogeneity among included trials, the Hartung-Knapp adjusted random-effects model was used for meta-analysis. The heterogeneity among studies was quantified using the *I*^2^ statistic and the between-study variance *τ*^2^. For outcomes with substantial heterogeneity, 95% prediction intervals (PIs) were reported to reflect the expected range of true treatment effects in future comparable studies. Dose-stratified subgroup analyses were performed to explore potential effect modification by ciprofol induction dose. Subgroups were defined as induction dose <0.4 mg/kg (low-dose group) and ≥0.4 mg/kg (high-dose group) [26]. To assess the robustness of the results, sensitivity analyses were performed using the leave-one-out method. In leave-one-out analysis, pooled results were considered unstable if exclusion of any single study altered the statistical significance of the outcome. Additionally, we used funnel plots for visual assessment of publication bias, and Egger’s and Begg’s tests were performed to evaluate publication bias statistically. Statistical significance was set at *p* < 0.05.

## 3. Results

### 3.1. Study Selection

**Figure 1:**
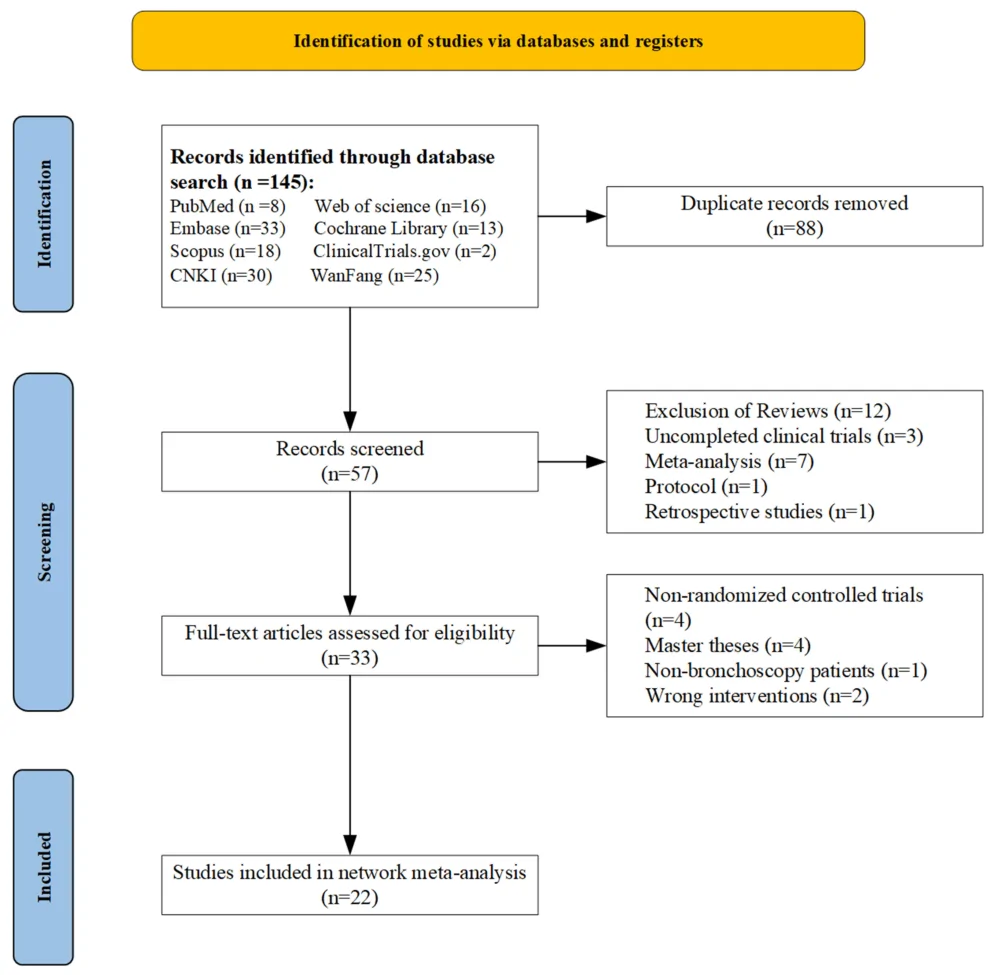
PRISMA 2020 flow diagram illustrating the study selection process for the systematic review and meta-analysis.

A total of 145 studies were retrieved from these databases. After removing duplicate titles and abstracts, 33 full-text articles were assessed for eligibility. Ultimately, 22 RCTs met the inclusion criteria and were included in this meta-analysis [27,28,29,30,31,32,33,34,35,36,37,38,39,40,41,42,43,44,45,46,47,48]. A detailed literature screening flow chart is shown in Figure 1.

### 3.2. Study Characteristics

All included RCTs were conducted in China and enrolled adult patients who underwent fiberoptic bronchoscopy. A total of 2452 participants were enrolled across these trials, with an overall male proportion of 54.73% and female proportion of 45.27%. The mean age of the included participants ranged from 36.8 to 71.9 years, and the mean BMI values varied between 21.4 and 25.3 kg/m^2^. Regarding trial design of included RCTs, only three studies were multicenter and eight studies performed double-blind design. Several trials lacked trial registration information and complete ASA-grade data. For studies with available ASA data, most participants belonged to ASA I–II grade, and few subjects were classified as ASA III. The characteristics of the included studies are summarized in Table S3. Table S4 summarizes the regimens of ciprofol and propofol regarding anesthetic induction, concomitant medications, and maintenance/rescue administration. Across the included studies, the induction dose of ciprofol was mostly 0.4 mg/kg, while the induction dose of propofol was generally 1.5 mg/kg.

### 3.3. Risk of Bias Assessment

**Figure 2:**
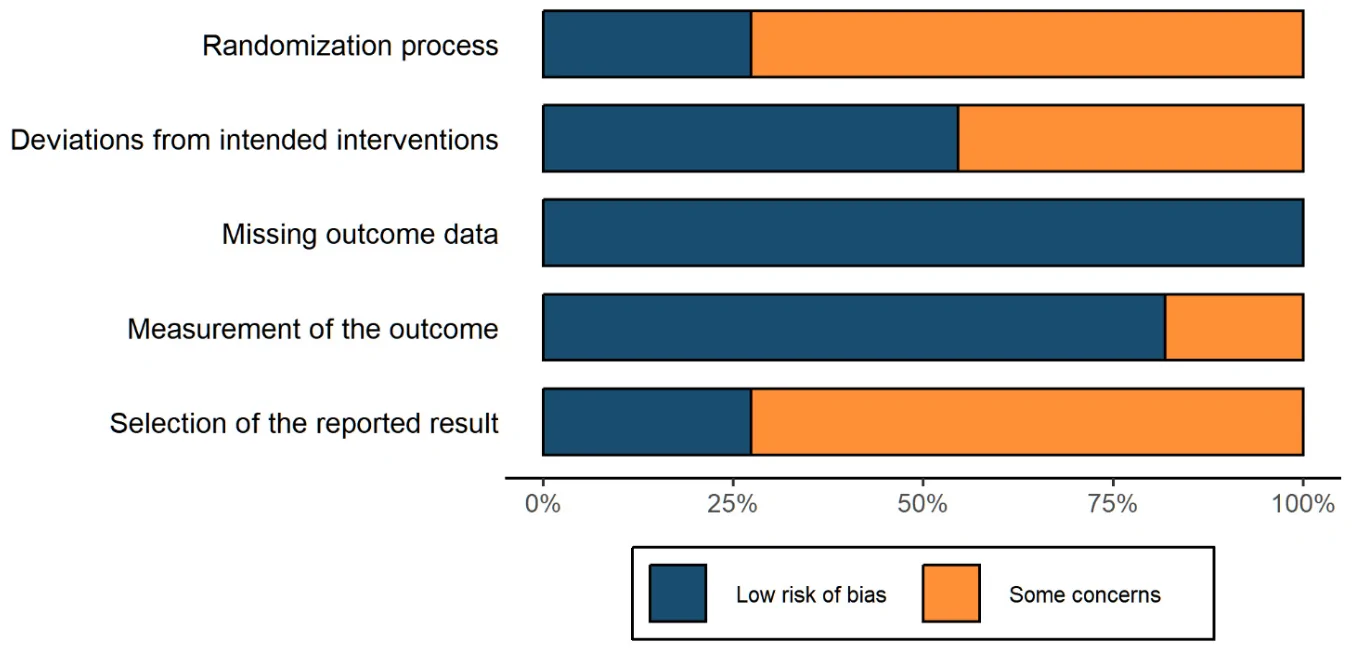
The risk of bias map depicting the proportion of each bias risk item in all studies.

**Figure 3:**
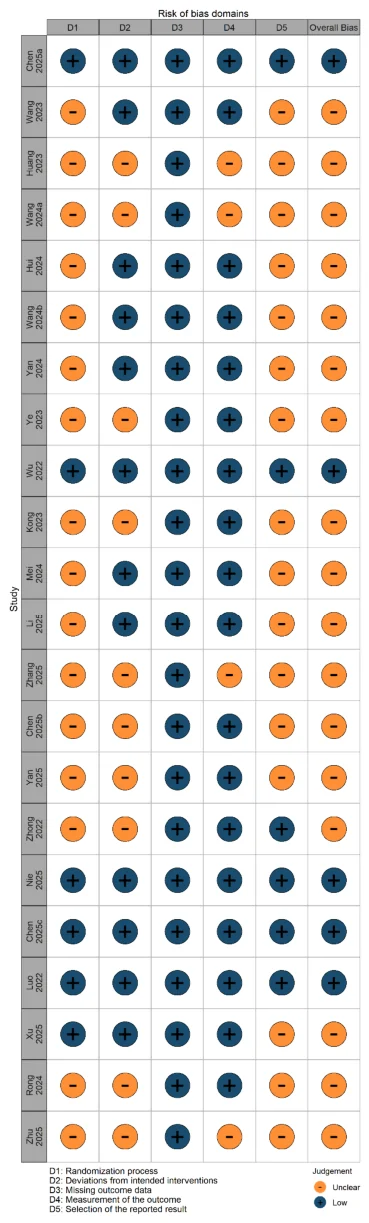
Risk of bias for included studies assessed by Cochrane risk of bias tool (ROB 2.0).

All included studies were assessed for risk of bias using the ROB 2.0 tool. In Domain 1 (Randomization process), 16 studies did not report allocation concealment and were rated as “some concerns” [28,29,30,31,32,33,34,36,37,38,39,40,41,42,47,48]. In Domain 2 (Deviations from intended intervention), 10 studies lacked detailed information on blinding implementation, leading to a rating of “some concerns” [29,30,34,36,39,40,41,42,47,48]. In Domain 5 (Selection of the reported result), 16 studies had no trial registration, making it impossible to verify consistency with the pre-specified protocol, thus rated as “some concerns” [28,29,30,31,32,33,34,36,37,38,39,40,41,46,47,48]. Overall, no high risk of bias was identified among the 22 included studies, whereas 17 studies [28,29,30,31,32,33,34,36,37,38,39,40,41,42,46,47,48] were considered to have “some concerns” (Figure 2 and Figure 3).

### 3.4. Adverse Events

#### 3.4.1. Hypotension

**Figure 4:**
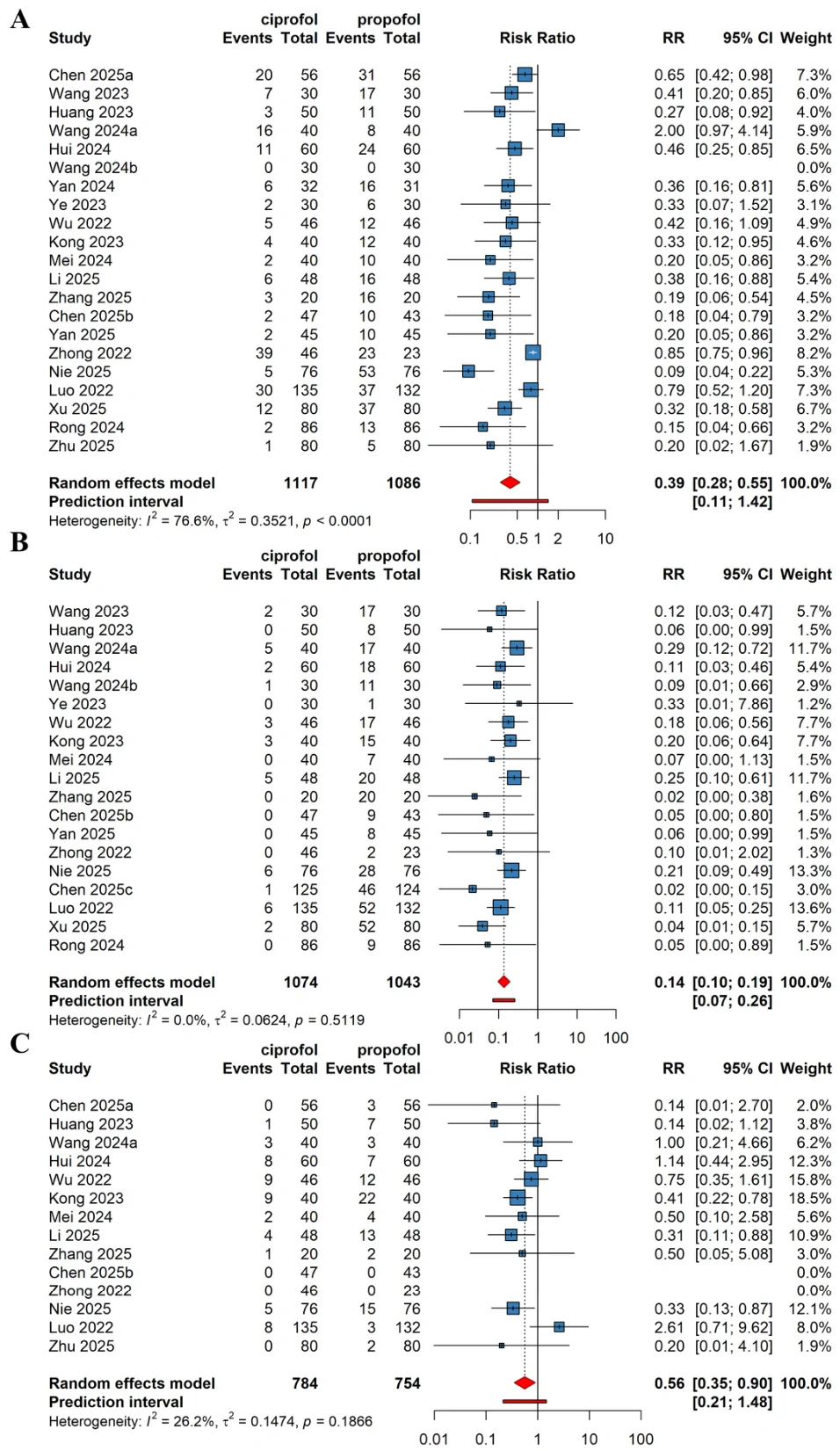
Forest plot for adverse event after administration of ciprofol compared with propofol: (**A**) Hypotension [27,28,29,30,31,32,33,34,35,36,37,38,39,40,41,42,43,45,46,47,48]. (**B**) Injection pain [28,29,30,31,32,34,35,36,37,38,39,40,41,42,43,44,45,46,47]. (**C**) Hypoxemia [27,29,30,31,35,36,37,38,39,40,42,43,45,48].

21 RCTs involving 2203 participants reported the incidence of hypotension [27,28,29,30,31,32,33,34,35,36,37,38,39,40,41,42,43,45,46,47,48]. Meta-analysis showed that ciprofol significantly reduced the risk of hypotension compared with propofol (RR, 0.39; 95% CI, 0.28–0.55). Substantial between-study heterogeneity was observed (*I*^2^ = 76.6%, *τ*^2^ = 0.3521), with a 95% PI of [0.11, 1.42] (Figure 4A).

#### 3.4.2. Injection Pain

19 RCTs (*N* = 2117) compared the incidence of injection pain [28,29,30,31,32,34,35,36,37,38,39,40,41,42,43,44,45,46,47]. The results demonstrated that ciprofol significantly reduced the risk of injection pain compared to propofol (RR, 0.14; 95% CI, 0.10–0.19), and the 95% PI was [0.07, 0.26]. Low heterogeneity was found (*I*^2^ = 0.0%, *τ*^2^ = 0.0624) (Figure 4B).

#### 3.4.3. Hypoxemia

The incidence of hypoxemia was compared across the 14 RCTs (*N* = 1538) [27,29,30,31,35,36,37,38,39,40,42,43,45,48]. The results demonstrated that ciprofol significantly reduced the risk of hypoxemia compared to propofol (RR, 0.56; 95% CI, 0.35–0.90), and the 95% PI was [0.21, 1.48]. Low heterogeneity was observed (*I*^2^ = 26.2%, *τ*^2^ = 0.1474) (Figure 4C).

#### 3.4.4. Cough

**Figure 5:**
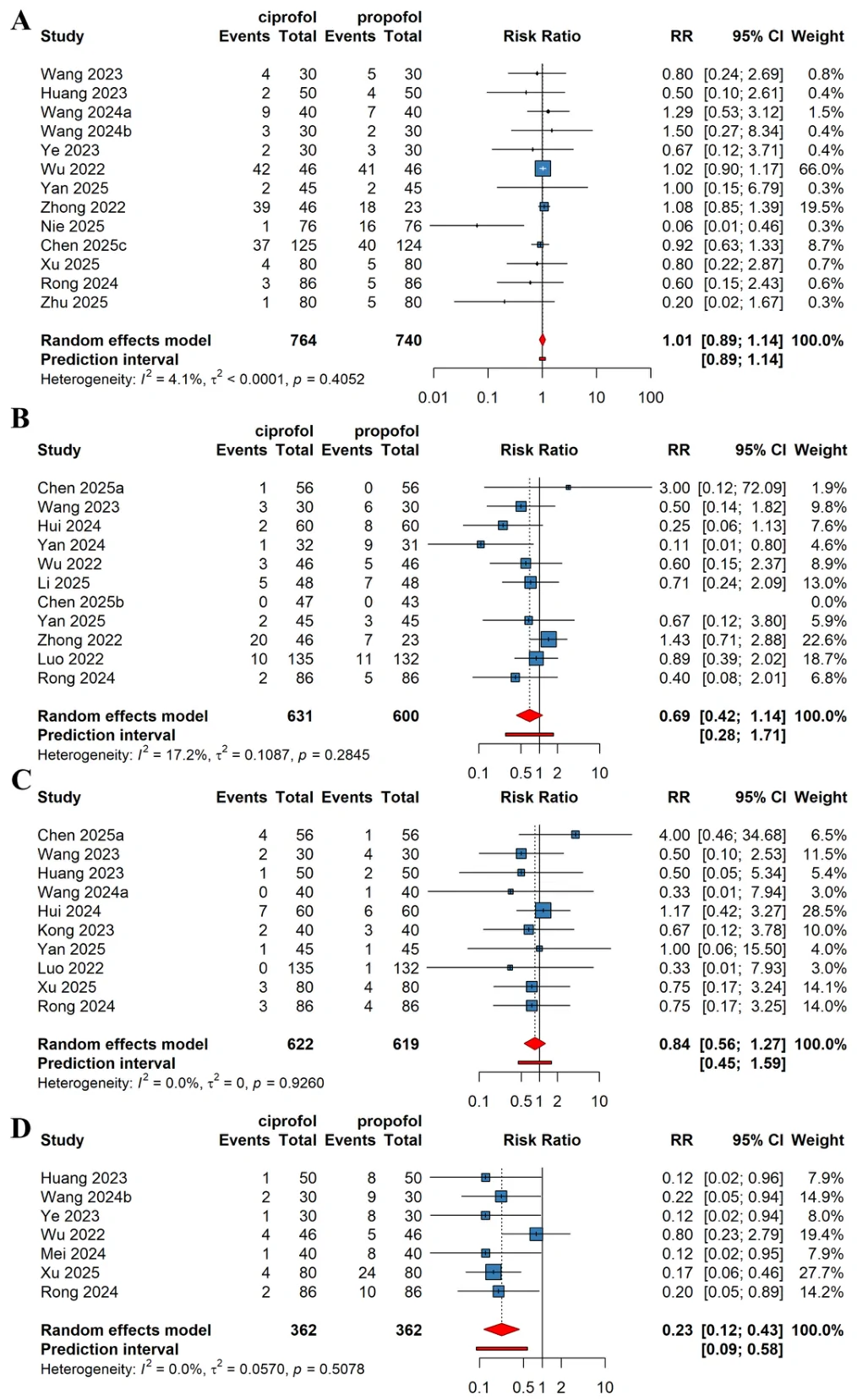
Forest plot for adverse event after administration of ciprofol compared with propofol: (**A**) Cough [28,29,30,32,34,35,41,42,43,44,46,47,48]. (**B**) Bradycardia [27,28,31,33,35,38,40,41,42,45,47]. (**C**) Nausea and vomiting [27,28,29,30,31,36,41,45,46,47]. (**D**) Respiratory depression sedation [29,32,34,35,37,46,47].

A meta-analysis of 13 RCTs, involving 1504 participants who underwent fiberoptic bronchoscopy, compared the incidence of cough between ciprofol and propofol sedation [28,29,30,32,34,35,41,42,43,44,46,47,48]. The results demonstrated that ciprofol had no significant difference in the risk of cough compared with propofol (RR, 1.01; 95% CI, 0.89–1.14), and the 95% PI was [0.89, 1.14]. Low heterogeneity was found (*I*^2^ = 4.1%, *τ*^2^ < 0.0001) (Figure 5A).

#### 3.4.5. Bradycardia

Eleven RCTs (*N* = 1231) were included in the bradycardia analysis [27,28,31,33,35,38,40,41,42,45,47]. The results demonstrated that ciprofol had no significant difference in the risk of bradycardia compared to propofol (RR, 0.69; 95% CI, 0.42–1.14), and the 95% PI was [0.28, 1.71]. Low heterogeneity was found (*I*^2^ = 17.2%, *τ*^2^ = 0.1087) (Figure 5B).

#### 3.4.6. Nausea and Vomiting

The incidence of nausea and vomiting was compared across the 10 RCTs (*N* = 1241) [27,28,29,30,31,36,41,45,46,47]. The results demonstrated that ciprofol had no significant difference in the risk of nausea and vomiting compared to propofol (RR, 0.84; 95% CI, 0.56–1.27), and the 95% PI was [0.45, 1.59]. No heterogeneity was found (*I*^2^ = 0.0%, *τ*^2^ = 0) (Figure 5C).

#### 3.4.7. Respiratory Depression

A meta-analysis of 7 RCTs involving 724 participants undergoing fiberoptic bronchoscopy, compared the incidence of respiratory depression between ciprofol and propofol sedation [29,32,34,35,37,46,47]. The results demonstrated that ciprofol significantly reduced the risk of respiratory depression compared to propofol (RR, 0.23; 95% CI, 0.12–0.43), and the 95% PI was [0.09, 0.58]. Low heterogeneity was found (*I*^2^ = 0.0%, *τ*^2^ = 0.0570) (Figure 5D).

### 3.5. Sedative Efficacy Outcomes

#### 3.5.1. Induction Time

**Figure 6:**
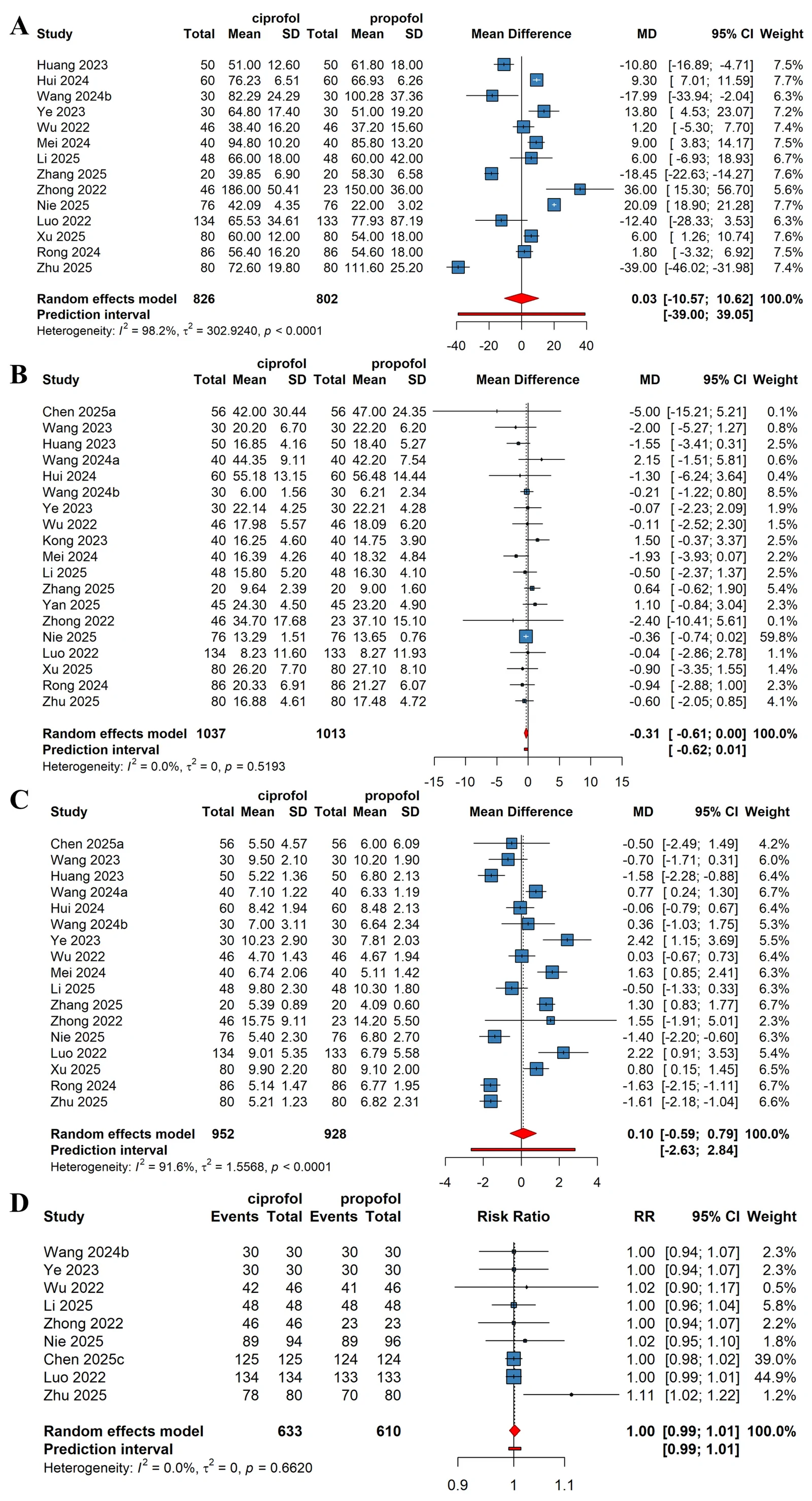
Forest plot for anesthetic effect with ciprofol versus propofol sedation: (**A**) Induction time (unit: seconds) [29,31,32,34,35,37,38,39,42,43,45,46,47,48]. (**B**) Operation time (unit: minutes) [27,28,29,30,31,32,34,35,36,37,38,39,41,42,43,45,46,47,48]. (**C**) Recovery time (unit: minutes) [27,28,29,30,31,32,34,35,37,38,39,42,43,45,46,47,48]. (**D**) Procedural success rate [32,34,35,38,42,43,44,45,48].

A meta-analysis of 14 RCTs involving 1628 participants undergoing fiberoptic bronchoscopy, compared the induction time between ciprofol and propofol sedation [29,31,32,34,35,37,38,39,42,43,45,46,47,48]. The results demonstrated that ciprofol had no significant difference in induction time compared to propofol (MD, 0.03 s; 95% CI, −10.57–10.62), with a 95% PI of [−39.00, 39.05]. Extremely high heterogeneity was observed (*I*^2^ = 98.2%, *τ*^2^ = 302.9240) (Figure 6A).

#### 3.5.2. Operation Time

Operation time was analyzed in 19 RCTs (*N* = 2050) [27,28,29,30,31,32,34,35,36,37,38,39,41,42,43,45,46,47,48]. The results demonstrated that ciprofol had no significant difference in operation time compared to propofol (MD, −0.3060 min; 95% CI, −0.6127–0.0008), with a 95% PI of [−0.62, 0.01]. No heterogeneity was found (*I*^2^ = 0.0%, *τ*^2^ = 0) (Figure 6B).

#### 3.5.3. Recovery Time

A meta-analysis of 17 RCTs involving 1880 participants undergoing fiberoptic bronchoscopy, compared the recovery time between ciprofol and propofol sedation [27,28,29,30,31,32,34,35,37,38,39,42,43,45,46,47,48]. The results demonstrated that ciprofol had no significant difference in recovery time compared to propofol (MD, 0.10 min; 95% CI, −0.59–0.79), with a 95% PI of [−2.63, 2.84]. Extremely high heterogeneity was observed (*I*^2^ = 91.6%, *τ*^2^ = 1.5568) (Figure 6C).

#### 3.5.4. Procedural Success Rate

Nine RCTs (*N* = 1243) evaluated the procedural success rate [32,34,35,38,42,43,44,45,48]. The results demonstrated that ciprofol had no significant difference in procedural success rate compared to propofol (RR, 1.00; 95% CI, 0.99–1.01), with a 95% PI of [0.99, 1.01]. No heterogeneity was found (*I*^2^ = 0.0%, *τ*^2^ = 0) (Figure 6D).

### 3.6. Subgroup Analysis

**Figure 7:**
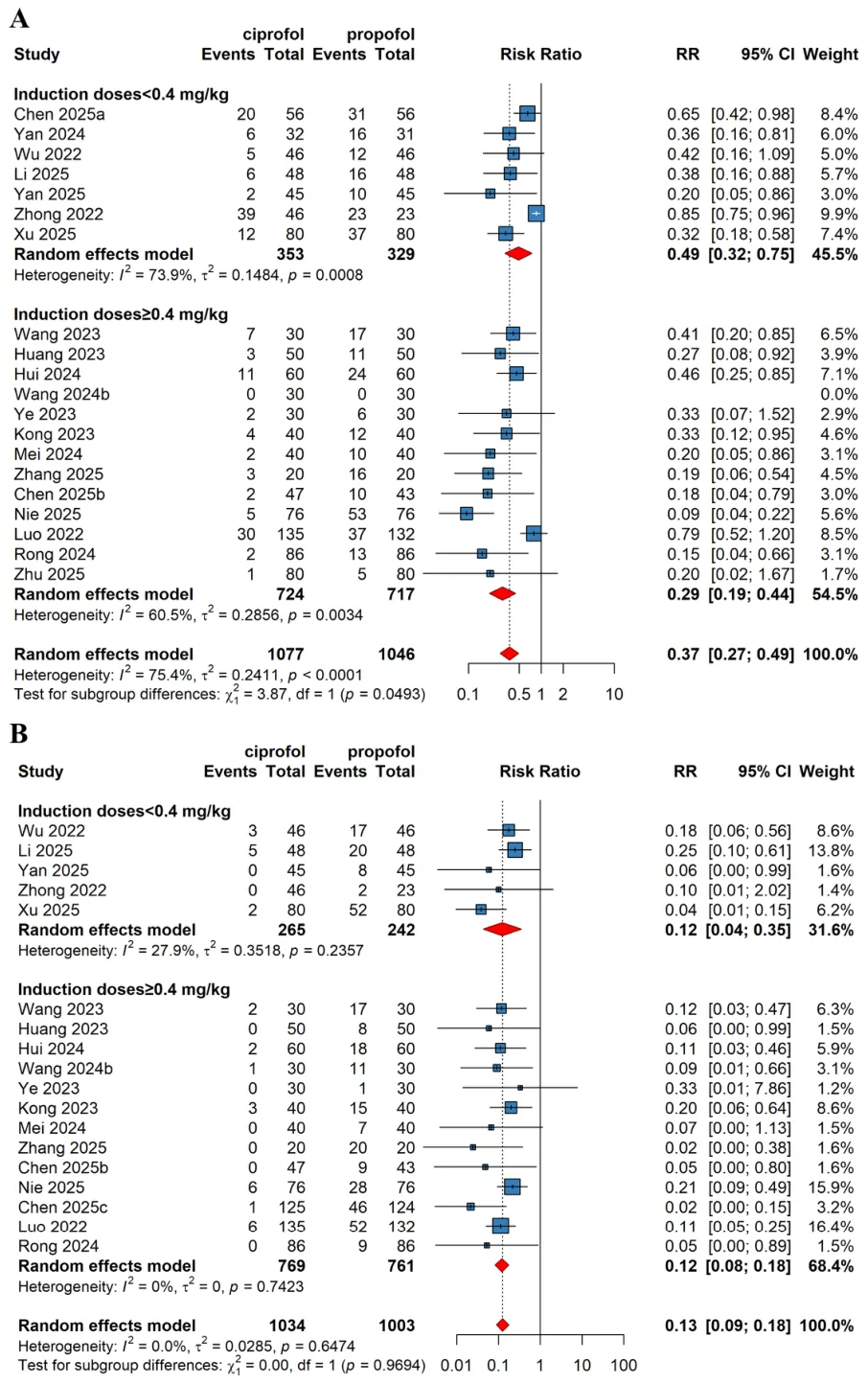
Forest plots of subgroup analysis based on ciprofol induction dose: (**A**) hypotension (studies were divided into the <0.4 mg/kg subgroup [27,33,35,38,41,42,46] and the ≥0.4 mg/kg subgroup [28,29,31,32,34,36,37,39,40,43,45,47,48]); (**B**) injection pain (studies were divided into the <0.4 mg/kg subgroup [35,38,41,42,46] and the ≥0.4 mg/kg subgroup [28,29,31,32,34,36,37,39,40,43,44,45,47]).

A subgroup analysis based on ciprofol induction dose (<0.4 mg/kg and ≥ 0.4 mg/kg) was conducted to explore the dose effect on the incidence of adverse events and sedative effect outcomes, with the results presented as follows. For adverse-event outcomes, statistically significant subgroup interaction was detected only for hypotension (*p* = 0.0493). The pooled RR was 0.49 (95% CI 0.32–0.75) for the <0.4 mg/kg subgroup and 0.29 (95% CI 0.19–0.44) for the ≥0.4 mg/kg subgroup (Figure 7A). There was no statistically significant interaction among other adverse event subgroups (Figure 7B and Figures S1–S5). Regarding sedative-efficacy outcomes, tests for subgroup differences were non-significant for induction time (*p* = 0.134), operation time (*p* = 0.626), recovery time (*p* = 0.858), and procedural success rate (*p* = 0.959) (Figures S6–S9).

### 3.7. Sensitivity Analysis

Figures S10–S20 summarize the results of the sensitivity analysis. Leave-one-out analysis demonstrated robust results for hypotension, injection pain, cough, nausea and vomiting, respiratory depression, induction time, recovery time and procedural success rate, as omitting any single trial did not change the statistical significance of pooled estimates. The results of the analysis of hypoxemia, bradycardia and operation time sensitivity were unstable. For hypoxemia, removal of Kong 2023 [36] or Nie 2025 [43] abolished the statistical significance of the pooled estimate. For bradycardia, excluding Zhong 2022 [42] shifted the result from non-significant to statistically significant. For operation time, omitting Wang 2024a [30], Kong 2023 [36], Zhang 2025 [39] or Yan 2025 [41] shifted the pooled result to statistical significance. Although these three outcomes were unstable, the direction of effect did not reverse after exclusion of influential studies; only the significance status changed.

### 3.8. Publication Bias

**Figure 8:**
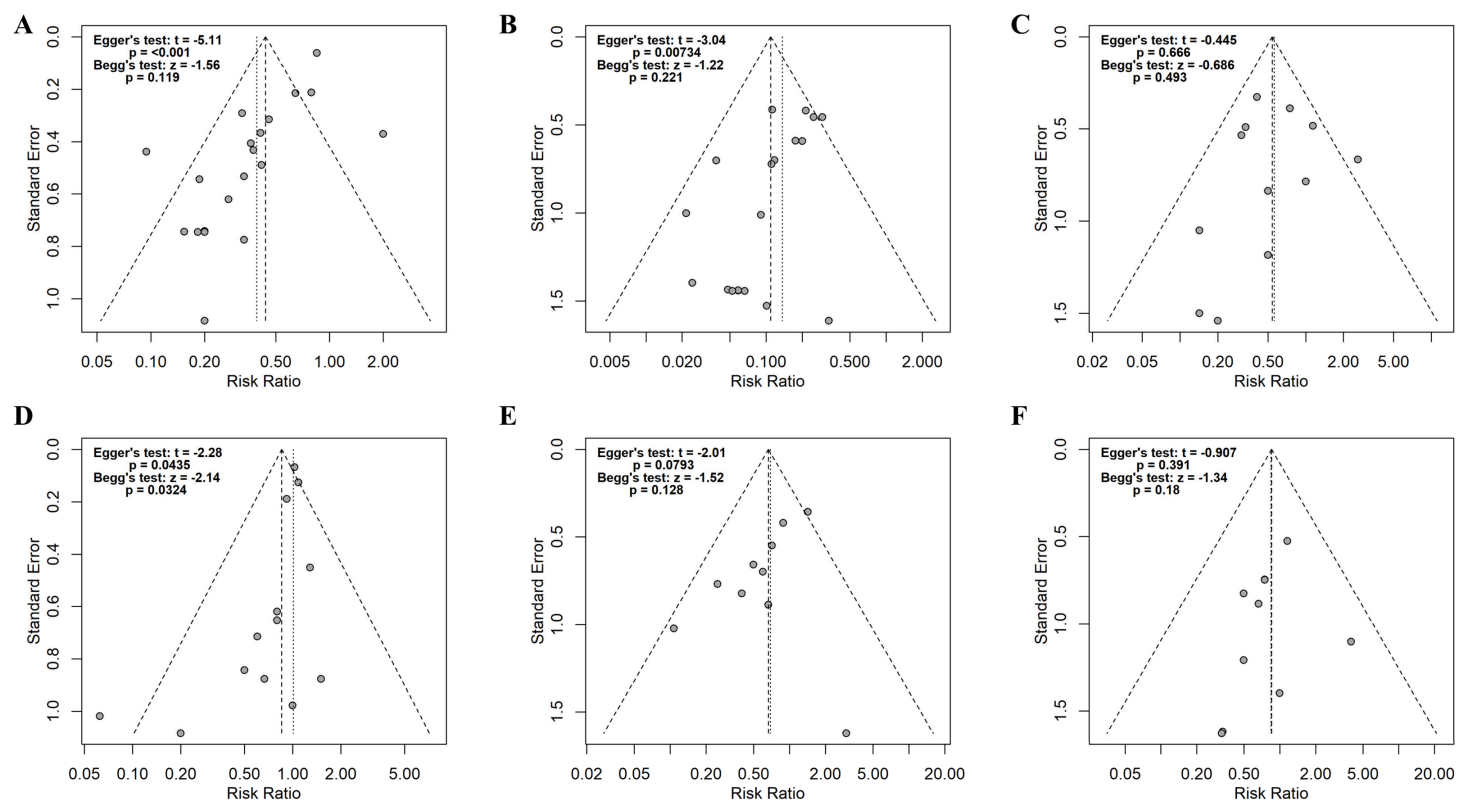
Assessment of publication bias for adverse event outcomes via funnel plot and statistical tests: (**A**) Hypotension. (**B**) Injection pain. (**C**) Hypoxemia. (**D**) Cough. (**E**) Bradycardia. (**F**) Nausea and vomiting.

Publication bias for the outcomes of adverse events and sedative efficacy was assessed using funnel plots combined with Egger’s and Begg’s tests. For adverse event outcomes, the funnel plot for hypotension and injection pain showed visual asymmetry, accompanied by significant Egger’s test (*p* < 0.05), while Begg’s test was non-significant. For cough, visual asymmetry was also observed, and the results of the Egger and Begg tests were both significant (*p* < 0.05) (Figure 8). For sedative efficacy outcomes, the funnel plot for induction time showed asymmetry, accompanied by significant Egger’s test (*p* < 0.05), while Begg’s test was non-significant (Figure S21).

Trim-and-fill analysis showed that the direction and significance of pooled estimates remained unchanged for hypotension (adjusted RR = 0.6669, 95% CI 0.4467–0.9958), injection pain (adjusted RR = 0.17, 95% CI 0.12–0.26) and cough (adjusted RR = 1.03, 95% CI 0.89–1.18), indicating robust results (Figures S22–S24). However, induction time became statistically significant after adjustment (adjusted MD = 14.75, 95% CI 1.46–28.05), suggesting substantial publication bias and warranting cautious interpretation (Figure S25).

### 3.9. Certainty of Evidence

The GRADE framework was applied to evaluate the certainty of evidence for 11 outcomes comparing ciprofol with propofol for sedation. The certainty of evidence was rated as moderate for injection pain, hypoxemia, bradycardia, respiratory depression and procedural success rate; low for hypotension, cough, nausea and vomiting, and operation time; and very low for induction time and recovery time (Table 1).

## 4. Discussion

This meta-analysis of 22 RCTs comprehensively evaluated the safety and efficacy of ciprofol versus propofol for sedation in patients undergoing fiberoptic bronchoscopy. The results demonstrated that ciprofol was associated with lower observed incidences of hypotension, injection pain, hypoxemia, and respiratory depression compared with propofol. No significant differences were observed between ciprofol and propofol regarding induction time, operation time, recovery time, or procedural success rate, indicating comparable sedative efficacy. Therefore, ciprofol may be a favorable alternative for sedation during fiberoptic bronchoscopy.

Regarding cardiovascular safety, this study indicated that ciprofol significantly reduced the risk of hypotension during fiberoptic bronchoscopy compared with propofol. This finding is consistent with the results of systematic reviews by Cheng et al. and Wang et al., which focused on gastrointestinal endoscopy [18,49]. The subgroup analysis indicated that at a dose of ≥0.4 mg/kg, ciprofol was more effective in reducing the risk of hypotension. This finding should be interpreted cautiously because the interaction *p*-value was close to 0.05. As this is an exploratory subgroup analysis, this dose-related observation requires further confirmation in future large-scale trials. The differences in cardiovascular safety between ciprofol and propofol may be attributed to their distinct pharmacological mechanisms. First, ciprofol exhibits a significantly higher binding affinity for γ-aminobutyric acid subtype A receptors (GABAARs) than propofol, requiring a lower dose to achieve equivalent sedative effects [50]. The reduced dosage directly alleviates the vasodilatory and negative inotropic effects on the cardiovascular system, thereby fundamentally reducing the risk of hypotension. Second, ciprofol has a rapid metabolic rate, and its metabolites are pharmacologically inactive, resulting in a shorter duration of cardiovascular inhibition than propofol [51]. From a clinical perspective, most patients receiving fiberoptic bronchoscopy are elderly. This group tolerates blood-pressure and cardiac-output fluctuations poorly. Studies have indicated that ciprofol has weaker inhibitory effects on myocardial contractility and peripheral vasculature, providing a more stable hemodynamic environment [52,53]. Consequently, ciprofol may be preferable for elderly patients and those with cardiovascular comorbidities.

In terms of respiratory safety, our study found that ciprofol significantly reduced the incidence of respiratory depression and hypoxemia compared with propofol. Studies conducted by Zeng et al. and Wang et al. reported consistent results [8,54]. This advantage can be explained by two mechanisms. Ciprofol exhibits higher sedative potency. It can produce equivalent sedation depth at lower doses and undergoes rapid in-vivo metabolism, which limits drug accumulation within the respiratory center [51]. Furthermore, there are differences in the selectivity of their actions on the GABA_A_ receptor subunits. Propofol has a high affinity for the β_2_ and β_3_ subunits of the GABA_A_ receptor. Its sedative effects are primarily mediated by these subunits, whereas respiratory depression is specifically regulated by the β_3_ subunit [55,56,57]. This suggests that ciprofol may act on distinct receptor subunits, thereby attenuating its inhibitory effect on the respiratory center. Thanks to this favorable pharmacodynamic feature, ciprofol may be better suited for several high-risk populations. These include patients with impaired respiratory function, the elderly, obese subjects, individuals with low baseline respiratory reserve, and patients sensitive to anesthetic-induced respiratory depression.

No significant difference in the risk of cough was observed between the two groups in this study, which was consistent with the findings of Zhou et al. (RR, 0.76; 95% CI, 0.53–1.10) [58]. Notably, among the 13 studies included in this analysis, only Nie et al. demonstrated a lower risk of cough with ciprofol. Currently, cough is rarely reported as an independent adverse event in bronchoscopy-related clinical studies, and the relevant clinical evidence is insufficient. Additional clinical data are needed for future analyses.

Furthermore, this meta-analysis found that the incidence of injection pain was significantly lower with ciprofol than with propofol alone. Injection pain is the most common local adverse reaction during propofol sedation, with an incidence as high as 20%, directly affecting patient comfort and the overall procedural experience [59]. The mechanism for propofol-induced injection pain has not been fully elucidated, but it may be associated with three potential pathways: Kallikrein-kinin system stimulation, activation of nociceptive transient receptor potential ion channels, and changes in osmotic pressure [60,61]. Injection pain may be related to the drug’s chemical structure and concentration. Ciprofol is modified from the molecular structure of propofol by introducing a cyclopropyl group, which significantly enhances its lipophilicity and hydrophobicity. This allows it to form a stable oil-in-water emulsion, with the free drug concentration in the aqueous phase being much lower than that of propofol [62]. Concurrently, its high lipophilicity enables rapid penetration of cell membranes and their distribution into peripheral tissues, further reducing the free drug concentration in local blood vessels [63]. Since aqueous free drug concentration is a key factor triggering injection pain, ciprofol can significantly reduce its occurrence [64]. Additionally, ciprofol has higher target selectivity and stronger in vitro and in vivo activities, with a sedative potency 4–5-fold higher than that of propofol. The lower dosage required further reduces the risk of local irritation.

Regarding indicators of sedative efficacy, including induction time, operation time, recovery time, and procedural success rate, no statistically significant differences were observed between ciprofol and propofol. Existing evidence on induction time is inconsistent. For instance, a meta-analysis by Zhou et al. reported that ciprofol had a slightly longer mean induction time than propofol, but the difference was not statistically significant (MD, 0.20; 95% CI, −0.22–0.63) [58]. In contrast, Wang et al. found that ciprofol had a slightly shorter induction time than propofol. Although the difference was statistically significant, high heterogeneity across studies precluded reliable conclusions (MD, −0.08; 95% CI, −0.24 to −0.08; *p* < 0.001; *I^2^* = 97.2%) [49]. This study also did not observe a significant difference in the induction time. Current evidence remains inconsistent, and further high-quality studies are required for validation. Regarding the surgical duration and procedural success rate, our results are consistent with those reported by Saseed et al., who showed no significant difference between ciprofol and propofol. Regarding recovery time, existing clinical evidence differs from our findings, possibly due to differences in study populations (Endoscopy vs. Bronchoscopy) and concomitant medication use [65]. Cheng et al.’s study on hysteroscopy reported that the ciprofol group had a longer recovery time than the propofol group [66]. A similar conclusion was documented in a meta-analysis of endoscopic diagnosis by Liu et al. [67]. Notably, our results on recovery time exhibited high heterogeneity (*I^2^* = 91.6%), which may be related to factors such as the dose-dependent metabolism of ciprofol and concomitant use of opioids such as remifentanil [68,69]. In summary, the overall sedative efficacies of ciprofol and propofol are comparable. In clinical practice, medication regimens should be adjusted based on individual patient characteristics and dosage to achieve optimal sedation.

Sensitivity analyses indicated that the meta-analysis results for most outcomes were robust, and exclusion of any single study did not alter the conclusions, supporting the reliability of the main findings of this review. However, the results for hypoxemia, bradycardia and operation time were unstable, suggesting that their pooled effects were driven by a few influential studies. Caution should therefore be exercised when interpreting these three endpoints. Despite these valuable insights, our study has several limitations. First, ciprofol is currently marketed in China. All 22 included RCTs were conducted in Chinese populations, lacking clinical data from other regions such as Europe and America. Meanwhile, East Asian populations have race-specific genetic polymorphisms in *UGT1A9*, the key metabolic enzyme for ciprofol, which may limit the global generalizability of the study results [70]. Second, most included studies did not provide detailed descriptions of bias control measures such as allocation concealment and blinding implementation. The varying methodological quality of the studies may have contributed to the increased heterogeneity of the results. Third, differences in the types and doses of concomitant opioid analgesics used during the procedures across studies may have introduced confounding bias, affecting the accuracy of the results. Fourth, the definition standards for some outcome indicators (e.g., recovery time and thresholds for adverse event determination) were not uniform across studies, leading to increased data heterogeneity and potentially affecting the reliability of the pooled results. Future high-quality, multicenter RCTs involving diverse populations, along with targeted pharmacological studies, are needed to further elucidate the efficacy and adverse event mechanisms of ciprofol.

## 5. Conclusions

This meta-analysis indicates that ciprofol shows lower observed risks of several adverse events relative to propofol for fiberoptic bronchoscopy, including hypotension, injection pain, hypoxemia, and respiratory depression, while maintaining comparable sedative efficacy. These findings suggest that ciprofol is a potentially safe alternative for bronchoscopic sedation. However, owing to geographic limitations and the generally limited methodological quality of the included studies, further multicenter RCTs are needed to validate these findings in diverse populations.

## Figures and Tables

**Table 1:** GRADE assessment of certainty of evidence for the comparison of ciprofol versus propofol for sedation.

Outcome	Participants (Studies)	Risk of Bias	Inconsistency	Indirectness	Imprecision	Others	RR/MD (95% CI)	Certainty of Evidence
Hypotension	2203 (21 RCTs)	not serious	serious	not serious	not serious	Serious ^a^	0.39 (0.28, 0.55)	⨁⨁◯◯ Low
Injection pain	2117 (19 RCTs)	not serious	not serious	not serious	not serious	Serious ^a^	0.14 (0.10, 0.19)	⨁⨁⨁◯ Moderate
Hypoxemia	1538 (14 RCTs)	not serious	not serious	not serious	serious	None	0.56 (0.35, 0.90)	⨁⨁⨁◯ Moderate
Cough	1504 (13 RCTs)	serious	not serious	not serious	not serious	Serious ^a^	1.01 (0.89, 1.14)	⨁⨁◯◯ Low
Bradycardia	1231 (11 RCTs)	not serious	not serious	not serious	serious	None	0.69 (0.42, 1.14)	⨁⨁⨁◯ Moderate
Nausea and vomiting	1241 (10 RCTs)	serious	not serious	not serious	serious	None	0.84 (0.56, 1.27)	⨁⨁◯◯ Low
Respiratory depression	724 (7 RCTs)	serious	not serious	not serious	not serious	None	0.23 (0.12, 0.43)	⨁⨁⨁◯ Moderate
Induction time	1628 (14 RCTs)	serious	very serious	not serious	serious	None	0.03 (−10.57, 10.62)	⨁◯◯◯ Very low
Operation time	2050 (19 RCTs)	serious	not serious	not serious	serious	None	−0.31 (−0.61, 0.00)	⨁⨁◯◯ Low
Recovery time	1880 (17 RCTs)	serious	serious	not serious	serious	None	0.10 (−0.59, 0.79)	⨁◯◯◯ Very low
Procedural success rate	1243 (9 RCTs)	serious	not serious	not serious	not serious	None	1.00 (0.99, 1.01)	⨁⨁⨁◯ Moderate

Notes: ^a^ publication bias strongly suspected. Abbreviations: GRADE, Grading of Recommendations Assessment, Development and Evaluation; RCT, randomised controlled trial; RR, risk ratio; MD, mean difference; CI, confidence interval. ⊕⊕⊕◯ Moderate certainty; ⊕⊕◯◯ Low certainty; ⊕◯◯◯ Very low certainty.

## Data Availability

The original contributions presented in this study are included in the article/Supplementary Material. Further inquiries can be directed to the corresponding authors.

## Supplementary Materials

The following supporting information can be downloaded at: https://www.mdpi.com/article/10.3390/ph19101575/s1, Table S1: PRISMA checklist; Table S2: Search strategy; Table S3: Characteristics of the included studies in this meta-analysis (*N* = 22); Table S4: Anesthetic regimens of included studies comparing ciprofol and propofol; Figure S1: Subgroup analysis of hypoxemia based on ciprofol dose; Figure S2: Subgroup analysis of cough based on ciprofol dose; Figure S3: Subgroup analysis of bradycardia a based on ciprofol dose; Figure S4: Subgroup analysis of nausea and vomiting based on ciprofol dose; Figure S5: Subgroup analysis of respiratory depression based on ciprofol dose; Figure S6: Subgroup analysis of anesthesia induction time based on ciprofol dose; Figure S7: Subgroup analysis of operation time based on ciprofol dose; Figure S8: Subgroup analysis of recovery time after surgery based on ciprofol dose; Figure S9: Subgroup analysis of procedural success rate based on ciprofol dose; Figure S10: Leave one out sensitivity analysis for hypotension outcome; Figure S11: Leave one out sensitivity analysis for injection pain outcome; Figure S12: Leave one out sensitivity analysis for hypoxemia outcome; Figure S13: Leave one out sensitivity analysis for cough outcome; Figure S14: Leave one out sensitivity analysis for bradycardia outcome; Figure S15: Leave one out sensitivity analysis for nausea and vomiting outcome; Figure S16: Leave one out sensitivity analysis for respiratory depression outcome; Figure S17: Leave one out sensitivity analysis for induction time outcome; Figure S18: Leave one out sensitivity analysis for operation time outcome; Figure S19: Leave one out sensitivity analysis for recovery time outcome; Figure S20: Leave one out sensitivity analysis for procedural success rate outcome; Figure S21: Assessment of publication bias for anesthetic effect via funnel plot and statistical tests: (A) Anesthesia induction time; (B) Operation time; (C) Recovery time; Figure S22: Trim-and-fill analysis of hypotension; Figure S23: Trim-and-fill analysis of injection pain; Figure S24: Trim-and-fill analysis of cough; Figure S25: Trim-and-fill analysis of induction time.
