# Supplementary material for: Comparative Efficacy and Safety of Ciprofol Versus Propofol in Adults Undergoing Fiberoptic Bronchoscopy: A Systematic Review and Meta-Analysis of Randomized Controlled Trials
Source: Pharmaceuticals (Basel). 2026 Oct 4;19(10):1575. doi: 10.3390/ph19101575 (PMC13708340; doi:10.3390/ph19101575)
Supplement: Supplementary file 1 [file pharmaceuticals-19-01575-s001.zip › pharmaceuticals-4511972-supplementary.pdf]

Supplementary Material

**Supplementary material**	**Page**
**Table S1.** PRISMA checklist	3
**Table S2.** Search strategy	5
**Table S3.** Characteristics of the included studies in this meta-analysis (N=22).	8
**Table S4.** Anesthetic regimens of included studies comparing ciprofol and propofol.	10
**Figure S1.** Subgroup analysis of hypoxemia based on ciprofol dose.	11
**Figure S2.** Subgroup analysis of cough based on ciprofol dose.	11
**Figure S3.** Subgroup analysis of bradycardia a based on ciprofol dose.	12
**Figure S4.** Subgroup analysis of nausea and vomiting based on ciprofol dose.	12
**Figure S5.** Subgroup analysis of respiratory depression based on ciprofol dose.	13
**Figure S6.** Subgroup analysis of anesthesia induction time based on ciprofol dose.	13
**Figure S7.** Subgroup analysis of operation time based on ciprofol dose.	14
**Figure S8.** Subgroup analysis of recovery time after surgery based on ciprofol dose.	14
**Figure S9.** Subgroup analysis of procedural success rate based on ciprofol dose.	15
**Figure S10.** Leave-one-out sensitivity analysis for hypotension outcome.	15
**Figure S11.** Leave-one-out sensitivity analysis for injection pain outcome.	16
**Figure S12.** Leave-one-out sensitivity analysis for hypoxemia outcome.	16
**Figure S13.** Leave-one-out sensitivity analysis for cough outcome.	17
**Figure S14.** Leave-one-out sensitivity analysis for bradycardia outcome.	17
**Figure S15.** Leave-one-out sensitivity analysis for nausea and vomiting outcome.	17
**Figure S16.** Leave-one-out sensitivity analysis for respiratory depression outcome.	18
**Figure S17.** Leave-one-out sensitivity analysis for induction time outcome.	18
**Figure S18.** Leave-one-out sensitivity analysis for operation time outcome.	19
**Figure S19.** Leave-one-out sensitivity analysis for recovery time outcome.	19
**Figure S20.** Leave-one-out sensitivity analysis for procedural success rate outcome.	20
**Figure S21.** Assessment of publication bias for anesthetic effect via funnel plot and statistical tests. (A) Anesthesia induction time; (B) Operation time; (C) Recovery time.	20

**Figure S22.** Trim-and-fill analysis of hypotension.	20
**Figure S23.** Trim-and-fill analysis of injection pain.	21
**Figure S24.** Trim-and-fill analysis of cough.	21
**Figure S25.** Trim-and-fill analysis of induction time.	21
**Reference**	22

Table S1. PRISMA checklist

Section and Topic	Item #	Checklist item	Location where item is reported
**TITLE**			
Title	1	Identify the report as a systematic review.	Page 1
**ABSTRACT**			
Abstract	2	See the PRISMA 2020 for Abstracts checklist.	Table S1
**INTRODUCTION**			
Rationale	3	Describe the rationale for the review in the context of existing knowledge.	Page 1-2
Objectives	4	Provide an explicit statement of the objective(s) or question(s) the review addresses.	Page 1-2
**METHODS**			
Eligibility criteria	5	Specify the inclusion and exclusion criteria for the review and how studies were grouped for the syntheses.	Page 2-4
Information sources	6	Specify all databases, registers, websites, organisations, reference lists and other sources searched or consulted to identify studies. Specify the date when each source was last searched or consulted.	Page 2-4
Search strategy	7	Present the full search strategies for all databases, registers and websites, including any filters and limits used.	Table S2
Selection process	8	Specify the methods used to decide whether a study met the inclusion criteria of the review, including how many reviewers screened each record and each report retrieved, whether they worked independently, and if applicable, details of automation tools used in the process.	Page 2-4
Data collection process	9	Specify the methods used to collect data from reports, including how many reviewers collected data from each report, whether they worked independently, any processes for obtaining or confirming data from study investigators, and if applicable, details of automation tools used in the process.	Page 2-4
Data items	10a	List and define all outcomes for which data were sought. Specify whether all results that were compatible with each outcome domain in each study were sought (e.g. for all measures, time points, analyses), and if not, the methods used to decide which results to collect.	Page 2-4
	10b	List and define all other variables for which data were sought (e.g. participant and intervention characteristics, funding sources). Describe any assumptions made about any missing or unclear information.	Page 2-4
Study risk of bias assessment	11	Specify the methods used to assess risk of bias in the included studies, including details of the tool(s) used, how many reviewers assessed each study and whether they worked independently, and if applicable, details of automation tools used in the process.	Page 2-4
Effect measures	12	Specify for each outcome the effect measure(s) (e.g. risk ratio, mean difference) used in the synthesis or presentation of results.	Page 2-4
Synthesis methods	13a	Describe the processes used to decide which studies were eligible for each synthesis (e.g. tabulating the study intervention characteristics and comparing against the planned groups for each synthesis (item #5)).	Page 2-4
	13b	Describe any methods required to prepare the data for presentation or synthesis, such as handling of missing summary statistics, or data conversions.	Page 2-4
	13c	Describe any methods used to tabulate or visually display results of individual studies and syntheses.	Page 2-4
	13d	Describe any methods used to synthesize results and provide a rationale for the choice(s). If meta-analysis was performed, describe the model(s), method(s) to identify the presence and extent of statistical heterogeneity, and software package(s) used.	Page 2-4
	13e	Describe any methods used to explore possible causes of heterogeneity among study results (e.g. subgroup analysis, meta-regression).	Page 2-4
	13f	Describe any sensitivity analyses conducted to assess robustness of the synthesized results.	Page 2-4
Reporting bias assessment	14	Describe any methods used to assess risk of bias due to missing results in a synthesis (arising from reporting biases).	Page 2-4
Certainty assessment	15	Describe any methods used to assess certainty (or confidence) in the body of evidence for an outcome.	Page 2-4
**RESULTS**			

Section and Topic	Item #	Checklist item	Location where item is reported
Study selection	16a	Describe the results of the search and selection process, from the number of records identified in the search to the number of studies included in the review, ideally using a flow diagram.	Page 4-5
	16b	Cite studies that might appear to meet the inclusion criteria, but which were excluded, and explain why they were excluded.	Figure 1
Study characteristics	17	Cite each included study and present its characteristics.	Page 4-5
Risk of bias in studies	18	Present assessments of risk of bias for each included study.	Page 5-6, Figures 3-3
Results of individual studies	19	For all outcomes, present, for each study: (a) summary statistics for each group (where appropriate) and (b) an effect estimate and its precision (e.g. confidence/credible interval), ideally using structured tables or plots.	Page 6
Results of syntheses	20a	For each synthesis, briefly summarise the characteristics and risk of bias among contributing studies.	Page 6-11
	20b	Present results of all statistical syntheses conducted. If meta-analysis was done, present for each the summary estimate and its precision (e.g. confidence/credible interval) and measures of statistical heterogeneity. If comparing groups, describe the direction of the effect.	Page 6-11
	20c	Present results of all investigations of possible causes of heterogeneity among study results.	Page 6-11
	20d	Present results of all sensitivity analyses conducted to assess the robustness of the synthesized results.	Page 6-11
Reporting biases	21	Present assessments of risk of bias due to missing results (arising from reporting biases) for each synthesis assessed.	Page 12
Certainty of evidence	22	Present assessments of certainty (or confidence) in the body of evidence for each outcome assessed.	Page 13, Table 1
**DISCUSSION**			
Discussion	23a	Provide a general interpretation of the results in the context of other evidence.	Page 13-16
	23b	Discuss any limitations of the evidence included in the review.	Page 15-16
	23c	Discuss any limitations of the review processes used.	Page 15-16
	23d	Discuss implications of the results for practice, policy, and future research.	Page 13-16
**OTHER INFORMATION**			
Registration and protocol	24a	Provide registration information for the review, including register name and registration number, or state that the review was not registered.	Page 2
	24b	Indicate where the review protocol can be accessed, or state that a protocol was not prepared.	Page 2
	24c	Describe and explain any amendments to information provided at registration or in the protocol.	Page 2
Support	25	Describe sources of financial or non-financial support for the review, and the role of the funders or sponsors in the review.	Page 16
Competing interests	26	Declare any competing interests of review authors.	Page 16
Availability of data, code and other materials	27	Report which of the following are publicly available and where they can be found: template data collection forms; data extracted from included studies; data used for all analyses; analytic code; any other materials used in the review.	Page 16

From: Page MJ, McKenzie JE, Bossuyt PM, Boutron I, Hoffmann TC, Mulrow CD, et al. The PRISMA 2020 statement: an updated guideline for reporting systematic reviews. BMJ 2021;372:n71. doi: 10.1136/bmj.n71

Table S2. Search strategy

(1) Search Strategy for PubMed

ID	Search	Results
#1	(Ciprofol[Title/Abstract]) OR (HSK3486[Title/Abstract])	219
#2	("Bronchoscopy"[Mesh] OR Bronchoscopies OR Bronchoscopic Surgical Procedures OR Bronchoscopic Surgical Procedure OR Bronchoscopic Surgery OR Surgery, Bronchoscopic OR Surgical Procedures, Bronchoscopic)	44029
#3	((((((((((Randomized Controlled Trial[Publication Type])) OR (Controlled Clinical Trial[Publication Type])) OR (randomized[Title/Abstract])) OR (randomly[Title/Abstract])) OR (random*[Title/Abstract])) OR (double-blind[Title/Abstract])) OR (single-blind[Title/Abstract])) OR (clinical trial*[Title/Abstract])) OR (trial*[Title/Abstract])) OR (RCT[Title/Abstract])) AND (humans[Filter])	1984323
#4	#1 and #2	12
#5	#1 and #2 and #3	8

(2) Search Strategy for Web of Science

ID	Search	Results
#1	(TS=(Ciprofol OR HSK3486)) NOT SO=(Preprint Citation Index)	313
#2	(TS= (Bronchoscopy OR bronchoscopes OR Bronchoscopic Surgical Procedures OR Bronchoscopic Surgical Procedure OR Bronchoscopic Surgery OR Surgery, Bronchoscopic OR Surgical Procedures, Bronchoscopic)) NOT SO=(Preprint Citation Index)	68657
#3	(TS= (Randomized Controlled Trial OR Controlled Clinical Trial OR randomized OR randomly OR random* OR double-blind OR single-blind OR clinical trial* OR trial* OR RCT AND humans)) NOT SO=(Preprint Citation Index)	6653500
#4	#1 AND #2 AND #3	16

(3) Search Strategy for EMBASE

ID	Search	Results

#1	(Ciprofol or HSK3486).ab,dm,dv,fx,hw,kf,mf,ot,ti,tn,dq.	580
#2	(Bronchoscopy or bronchoscopes or "Bronchoscopic Surgical Procedures" or "Bronchoscopic Surgical Procedure" or "Bronchoscopic Surgery" or "Surgery, Bronchoscopic" or "Surgical Procedures, Bronchoscopic").ab,dm,dv,fx,hw,kf,mf,ot,ti,tn,dq.	145663
#3	(Randomized Controlled Trial or "Controlled Clinical Trial" or randomized or randomly or random* or "double-blind" or "single-blind" or "clinical trial*" or trial* or RCT).ab,dm,dv,fx,hw,kf,mf,ot,ti,tn,dq.	9008134
#4	#1 and #2 and #3	33

(4) Search Strategy for Cochrane Library

ID	Search	Results
#1	Title Abstract Keyword (Ciprofol OR HSK3486)	473
#2	Title Abstract Keyword (Bronchoscopy OR Bronchoscopies OR Bronchoscopic Surgical Procedures OR Bronchoscopic Surgical Procedure OR Bronchoscopic Surgery OR Surgery, Bronchoscopic OR Surgical Procedures, Bronchoscopic)	3866
#3	Title Abstract Keyword (Randomized Controlled Trial OR Controlled Clinical Trial OR randomized OR randomly OR random* OR double-blind OR single-blind OR clinical trial* OR trial* OR RCT)	1661751
#4	#1 and #2 and #3	13

(5) Search Strategy for Scopus

ID	Search	Results
#1	TITLE-ABS-KEY ("Ciprofol" OR "HSK3486")	250
#2	TITLE-ABS-KEY ("Bronchoscopy" OR "Bronchoscopies" OR "Bronchoscopic Surgical Procedures" OR "Bronchoscopic Surgical Procedure" OR "Bronchoscopic Surgery" OR "Surgery, Bronchoscopic" OR "Surgical Procedures, Bronchoscopic")	78345

#3	TITLE-ABS-KEY ("Randomized Controlled Trial" OR "Controlled Clinical Trial" OR "randomized" OR "randomly" OR "random*" OR "double-blind" OR "single-blind" OR "clinical trial*" OR "trial*" OR "RCT")	5706707
#4	#1 and #2 and #3	18

(6) Search Strategy for CNKI

ID	Search	Results
#1	Ciprofol(in Chinese)	485
#2	Bronchoscopy(in Chinese)	31470
#3	#1 and #2	30

(7) Search Strategy for Wanfang database

ID	Search	Results
#1	Ciprofol(in Chinese precise)	561
#2	Bronchoscopy	32089
#3	#1 and #2	25

(8) Search Strategy for Clinical trials

ID	Search	Results
#1	"Ciprofol" OR "HSK3486"	82
#2	"Bronchoscopy" OR "Bronchoscopies" OR "Bronchoscopic Surgical Procedures" OR "Bronchoscopic Surgical Procedure" OR "Bronchoscopic Surgery" OR "Surgery, Bronchoscopic" OR "Surgical Procedures, Bronchoscopic"	1367
#3	("Randomized Controlled Trial" OR "Controlled Clinical Trial" OR "randomized" OR "randomly" OR "random*" OR "double-blind" OR "single-blind" OR "clinical trial*" OR "trial*" OR "RCT")	353615
#4	#1 and #2	2
#5	#1 and #2 and #3	2

Table S3. Characteristics of studies included in this meta-analysis (N=22).

Study ID	Country	Trial number	Study design	Mask	Study period	Participants	Intervention (experimental/control)	Gender (male/female)	Ages (years, mean±sd)	BMI (kg/m2, mean±sd)	ASA classification (I/II/III)
Chen 2025a[1]	China	ChiCTR2200062721	multicenter	double-blind	2022.11-2023.06	112	ciprofol<br>propofol	24/32<br>27/29	62.0±13.0<br>64.0±10.0	24.0±4.0<br>25.0±4.0	0/41/15<br>0/42/14
Wang 2023[2]	China	NA	single-center	NA	2022.12-2023.03	60	ciprofol<br>propofol	13/17<br>14/16	60.1±14.6<br>59.0±15.8	25.3±2.1<br>24.3±3.5	11/19/0<br>7/23/0
Huang 2023[3]	China	NA	single-center	NA	2022.08-2022.09	100	ciprofol<br>propofol	30/20<br>28/22	43.7±5.0<br>44.2±5.2	NA	35/15/0<br>33/17/0
Wang 2024a[4]	China	NA	single-center	NA	2023.05-2023.10	80	ciprofol<br>propofol	29/11<br>30/10	61.4±8.1<br>61.1±7.9	23.9±2.0<br>23.1±2.4	4/31/5<br>4/31/5
Hui 2024[5]	China	NA	single-center	double-blind	2022.10-2023.03	120	ciprofol<br>propofol	39/21<br>41/18	69.8±7.2<br>69.6±7.2	23.4±2.7<br>23.5±2.7	28/32/0<br>24/36/0
Wang 2024b[6]	China	NA	single-center	NA	2022.10-2023.01	60	ciprofol<br>propofol	15/15<br>22/8	71.3±22.2<br>71.9±17.7	NA	NA
Yan 2024[7]	China	NA	single-center	NA	2023.11-2024.01	63	ciprofol<br>propofol	11/21<br>18/13	44.7±16.4<br>45.5±16.2	22.8±3.9<br>21.4±3.1	NA
Ye 2023[8]	China	NA	single-center	NA	2021.01-2023.03	60	ciprofol<br>propofol	13/17<br>14/16	52.6±7.4<br>49.8±8.3	23.2±2.2<br>22.6±2.3	6/23/1<br>5/24/1
Wu 2022[9]	China	ChiCTR2200062763	single-center	double-blind	2022.08-2022.11	92	ciprofol<br>propofol	26/20<br>24/22	58.0±5.5<br>57.5±5.3	24.3±1.8<br>24.5±2.1	10/36/0<br>8/38/0
Kong 2023[10]	China	NA	single-center	NA	2022.01-2022.12	80	ciprofol<br>propofol	26/14<br>23/17	67.2±9.6<br>64.4±8.8	23.1±3.1<br>23.5±3.2	31/9/0<br>29/11/0
Mei 2024[11]	China	NA	single-center	NA	2023.01-2023.12	80	ciprofol<br>propofol	27/13<br>25/15	58.8±3.4<br>59.5±3.2	NA	NA
Li 2025[12]	China	NA	single-center	NA	2024.03-2024.12	96	ciprofol<br>propofol	26/22<br>28/20	71.6±3.4<br>70.9±2.7	24.6±2.8<br>23.8±3.1	0/13/35<br>0/16/32
Zhang 2025[13]	China	ChiCTR2400087524	single-center	NA	2024.08-2024.12	40	ciprofol<br>propofol	9/11<br>14/6	36.8±17.2<br>46.7±18.2	21.9±1.7<br>21.5±1.1	8/12/0<br>9/11/0
Chen 2025b[14]	China	NA	single-center	NA	2023.03-2023.05	90	ciprofol<br>propofol	17/30<br>18/25	45.1±18.2<br>46.1±17.1	22.8±3.1<br>22.2±4.0	NA
Yan 2025[15]	China	NA	single-center	NA	2023.10-2024.10	90	ciprofol<br>propofol	24/21<br>23/22	46.5±4.8<br>46.9±4.9	23.1±2.5<br>22.9±2.3	NA

**Zhong 2022[16]**	China	ChiCTR2100051114	single-center	double-blind	2021.10-2021.12	69	ciprofol<br>propofol	19/27<br>11/12	55.7±15.6<br>55.8±12.6	22.5±3.2<br>22.0±2.3	5/37/4<br>4/17/2
**Nie 2025[17]**	China	ChiCTR2400081603	multicenter	double-blind	2024.03-2024.08	152	ciprofol<br>propofol	46/30<br>45/31	55.2±14.9<br>54.4±11.3	22.9±2.9<br>22.0±2.9	3/72/0<br>2/74/0
**Chen 2025c[18]**	China	ChiCTR2200063048	single-center	double-blind	2022.09-2023.06	249	ciprofol<br>propofol	73/52<br>71/53	62.7±12.8<br>62.9±11.8	22.2±5.3<br>23.0±3.5	0/102/23<br>0/102/22
**Luo 2022[19]**	China	NCT04111159	multicenter	double-blind	2019.12 - 2020.06	267	ciprofol<br>propofol	62/72<br>73/60	46.6±15.3<br>46.9±14.0	23.2±2.6<br>22.9±2.6	63/68/3<br>50/82/1
**Xu 2025[20]**	China	NA	single-center	double-blind	2024.04-2024.12	160	ciprofol<br>propofol	39/41<br>40/40	60.1±5.7<br>59.7±6.1	22.1±2.6<br>21.9±2.4	4/76/0<br>5/75/0
**Rong 2024[21]**	China	NA	single-center	NA	2023.01-2023.12	172	ciprofol<br>propofol	47/39<br>49/37	52.4±3.8<br>52.4±3.7	21.4±2.1<br>21.4±2.1	51/35/0<br>53/33/0
**Zhu 2025[22]**	China	NA	single-center	NA	2023.01-2024.12	160	ciprofol<br>propofol	45/35<br>43/37	55.8±5.2<br>55.8±5.2	21.8±1.2<br>21.8±1.1	NA

BMI, body mass index; ASA, American Society of Anesthesiologists Score; NA, not available;

Table S4. Anesthetic regimens of included studies comparing ciprofol and propofol.

Study	Arm	Induction dose (mg·kg⁻¹)	Concomitant medication	Dose*	Maintenance / rescue regimen
**Chen 2025a**	Ciprofol	0.1–0.3	Remifentanil	2–4 (TCI)	Ciprofol 0.4–1.2
	Propofol	1–3	Remifentanil	2–4 (TCI)	Propofol 4–12
**Wang 2023**	Ciprofol	0.4	Remifentanil	1	Ciprofol 0.8–1.2
	Propofol	2.5	Remifentanil	1	Propofol 4–6
**Huang 2023**	Ciprofol	0.4	Sufentanil	0.1	Rescue bolus: ciprofol 5 mg PRN
	Propofol	1.5	Sufentanil	0.1	Rescue bolus: propofol 20 mg PRN
**Wang 2024a**	Ciprofol	NA	Remifentanil	6–12 (inf.)	Ciprofol 0.6–1.2
	Propofol	NA	Remifentanil	6–12 (inf.)	Propofol 3–6
**Hui 2024**	Ciprofol	0.4	Alfentanil	20	Ciprofol 0.8 + alfentanil 0.5–1.0
	Propofol	2	Alfentanil	20	Propofol 8 + alfentanil 0.5–1.0 (IV infusion)
**Wang 2024b**	Ciprofol	0.4	Fentanyl	1	Rescue bolus: ciprofol 0.2 PRN
	Propofol	1.5	Fentanyl	1	Rescue bolus: propofol 0.5 PRN
**Yan 2024**	Ciprofol	0.3	Remifentanil	0.8	NA
	Propofol	1.2	Remifentanil	0.8	NA
**Ye 2023**	Ciprofol	0.4	Sufentanil	0.1	Rescue bolus: ciprofol 0.1–0.2 PRN
	Propofol	1.5–2.0	Sufentanil	0.1	Rescue bolus: propofol 0.2–0.5 PRN
**Wu 2022**	Ciprofol	0.3	Fentanyl	50 (fixed)	Rescue bolus: 1/3–1/4 of initial dose PRN
	Propofol	1.2	Fentanyl	50 (fixed)	Rescue bolus: 1/3–1/4 of initial dose PRN
**Kong 2023**	Ciprofol	0.4	Remifentanil	0.02	Ciprofol 1 + remifentanil 0.2
	Propofol	1.5	Remifentanil	0.02	Propofol 5 + remifentanil 0.2
**Mei 2024**	Ciprofol	0.4	Sufentanil	0.1	Rescue bolus: ciprofol 5 mg PRN
	Propofol	1.5	Sufentanil	0.1	Rescue bolus: propofol 20 mg PRN
**Li 2025**	Ciprofol	0.3	Sufentanil	0.1	Rescue bolus: ciprofol 0.15 PRN
	Propofol	1.5	Sufentanil	0.1	Rescue bolus: propofol 0.5–1.0 PRN
**Zhang 2025**	Ciprofol	0.4	Remifentanil	0.5	Rescue bolus: 1/3 of initial dose PRN
	Propofol	2	Remifentanil	0.5	Rescue bolus: 1/3 of initial dose PRN
**Chen 2025b**	Ciprofol	0.4	Sufentanil	0.1	Rescue bolus: 25%–33% of initial dose PRN
	Propofol	2	Sufentanil	0.1	Rescue bolus: 25%–33% of initial dose PRN
**Yan 2025**	Ciprofol	0.3–0.4	Remifentanil	0.5–1.0	Ciprofol 0.3–1.0 + remifentanil 0.05–0.10
	Propofol	1.0–1.5	Remifentanil	0.5–1.0	Propofol 1.5–4.5 + remifentanil 0.05–0.10
**Zhong 2022**	Ciprofol	0.3–0.4	Remifentanil	4 (TCI)	Ciprofol 1.0–2.5 + remifentanil 0.5–1.0 (TCI)
	Propofol	2	Remifentanil	4 (TCI)	Propofol 5–12.5 + remifentanil 0.5–1.0 (TCI)
**Nie 2025**	Ciprofol	0.4	Remifentanil	1	Rescue bolus: ciprofol 0.1–0.2 PRN
	Propofol	2.5	Remifentanil	1	Rescue bolus: propofol 0.2–0.5 PRN
**Chen 2025c**	Ciprofol	0.4	Sufentanil	0.2	Ciprofol 0.8–1.2 + sufentanil 0.05–2
	Propofol	2	Sufentanil	0.2	Propofol 4–12 + sufentanil 0.05–2
**Luo 2022**	Ciprofol	0.4	Sufentanil	0.2	Rescue bolus: ciprofol 0.15 PRN
	Propofol	2.0	Sufentanil	0.2	Rescue bolus: propofol 0.75 PRN
**Xu 2025**	Ciprofol	0.3	Esketamine	0.2 mg·kg⁻¹	Rescue bolus: ciprofol 0.15 PRN
	Propofol	1.5	Esketamine	0.2 mg·kg⁻¹	Rescue bolus: propofol 0.5–1.0 PRN
**Rong 2024**	Ciprofol	0.4	Sufentanil	0.1	Rescue bolus: ciprofol 5 mg PRN
	Propofol	1.5	Sufentanil	0.1	Rescue bolus: propofol 20 mg PRN
**Zhu 2025**	Ciprofol	0.4	Sufentanil	0.1	NA
	Propofol	1.5	Sufentanil	0.1	NA

NA, not available/reported; PRN, as needed (pro re nata); TCI, target-controlled infusion; inf., continuous infusion; IV, intravenous.
***Dose column:** Bolus/induction doses are expressed as $mg\cdot kg^{-1}$. Continuous maintenance infusions of ciprofol/propofol are expressed as $mg\cdot kg^{-1}\cdot h^{-1}$, and adjunct opioid infusions (alfentanil/remifentanil/sufentanil) are expressed as $\mu g\cdot kg^{-1}\cdot min^{-1}$.

For most studies the concomitant opioid was given as a bolus in $\mu g\cdot kg^{-1}$. Exceptions: Chen 2025a and Zhong 2022 used remifentanil as a TCI target concentration ($ng\cdot mL^{-1}$); Wang 2024a used remifentanil as a continuous infusion (6–12 $\mu g\cdot kg^{-1}\cdot h^{-1}$); Wu 2022 used fentanyl as a fixed total dose (50 μg); Xu 2025 used esketamine (an NMDA antagonist, not an opioid) at 0.2 $mg\cdot kg^{-1}$. Rescue boluses were administered at the investigators' discretion to suppress intraoperative movement or hemodynamic responses.

Figure S1. Subgroup analysis of hypoxemia based on ciprofol dose.

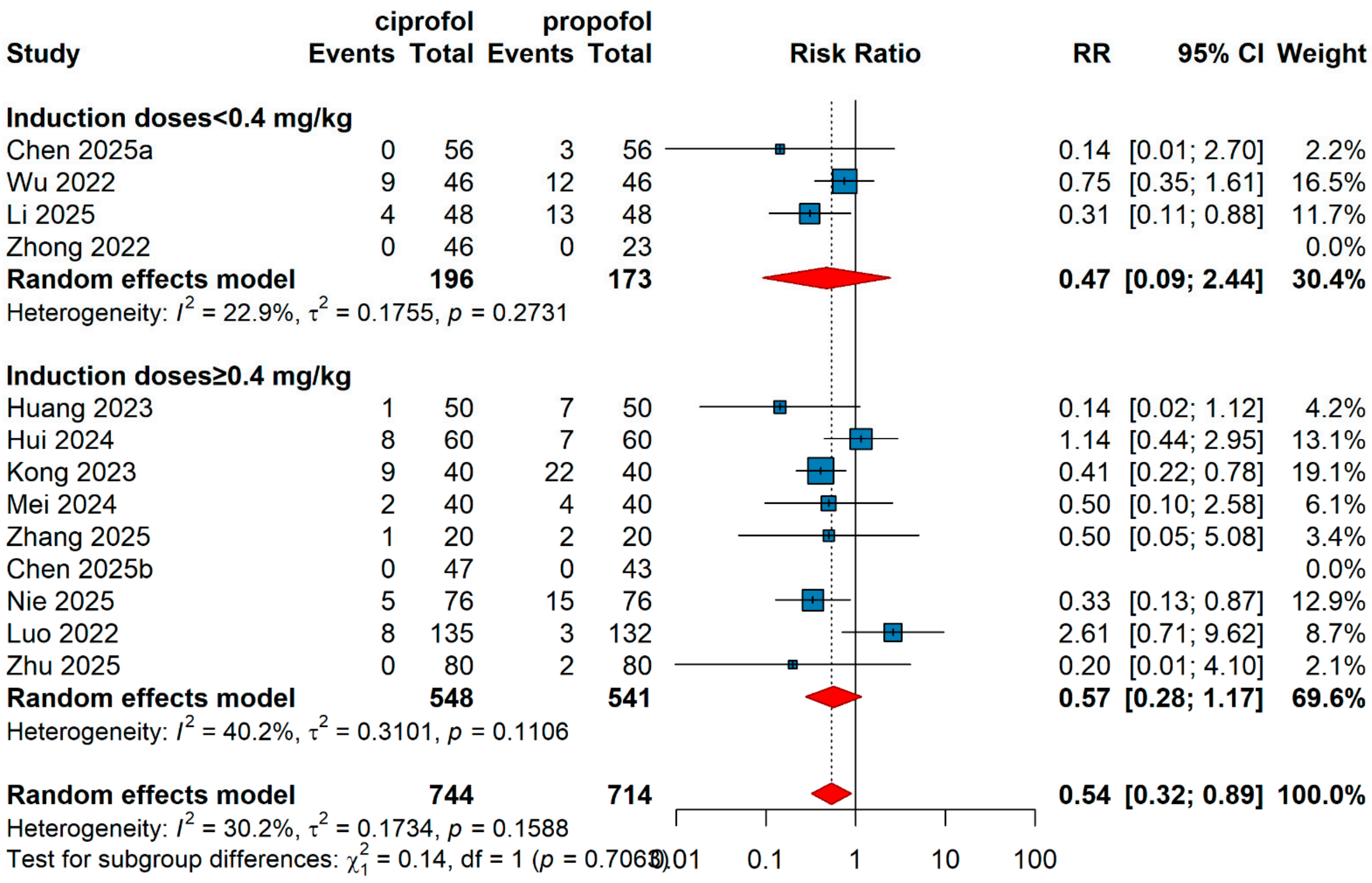

Figure S2. Subgroup analysis of cough based on ciprofol dose.

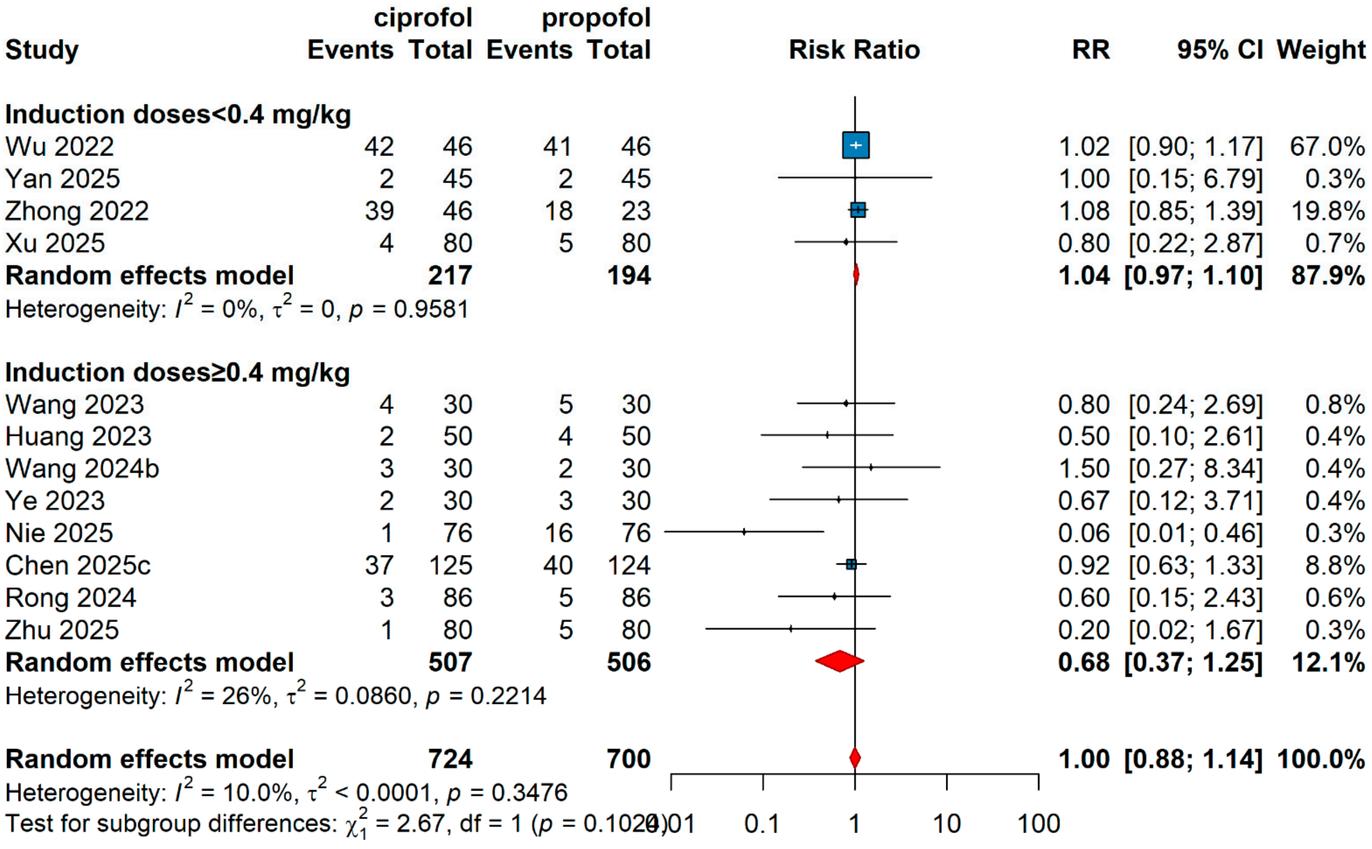

Figure S3. Subgroup analysis of bradycardia based on ciprofol dose.

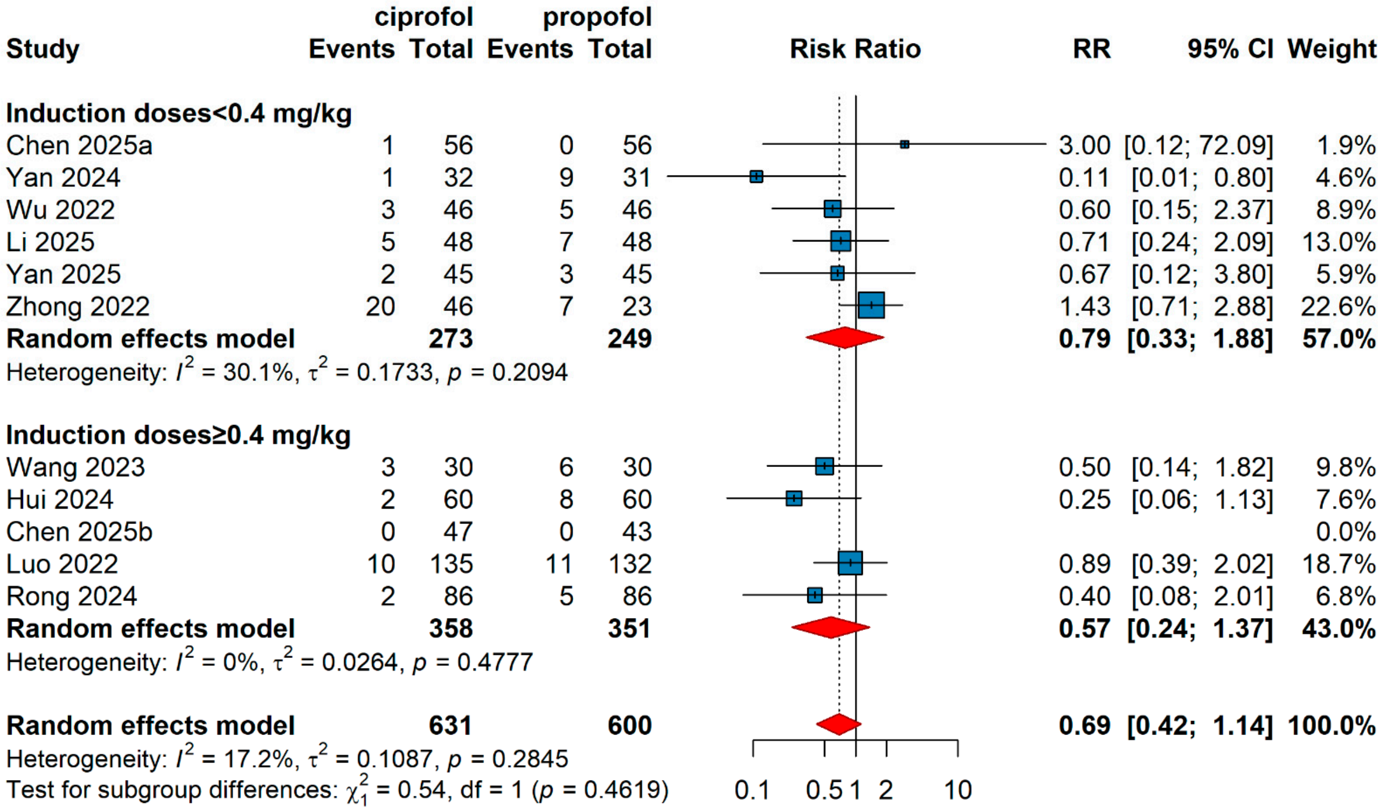

Figure S4. Subgroup analysis of nausea and vomiting based on ciprofol dose.

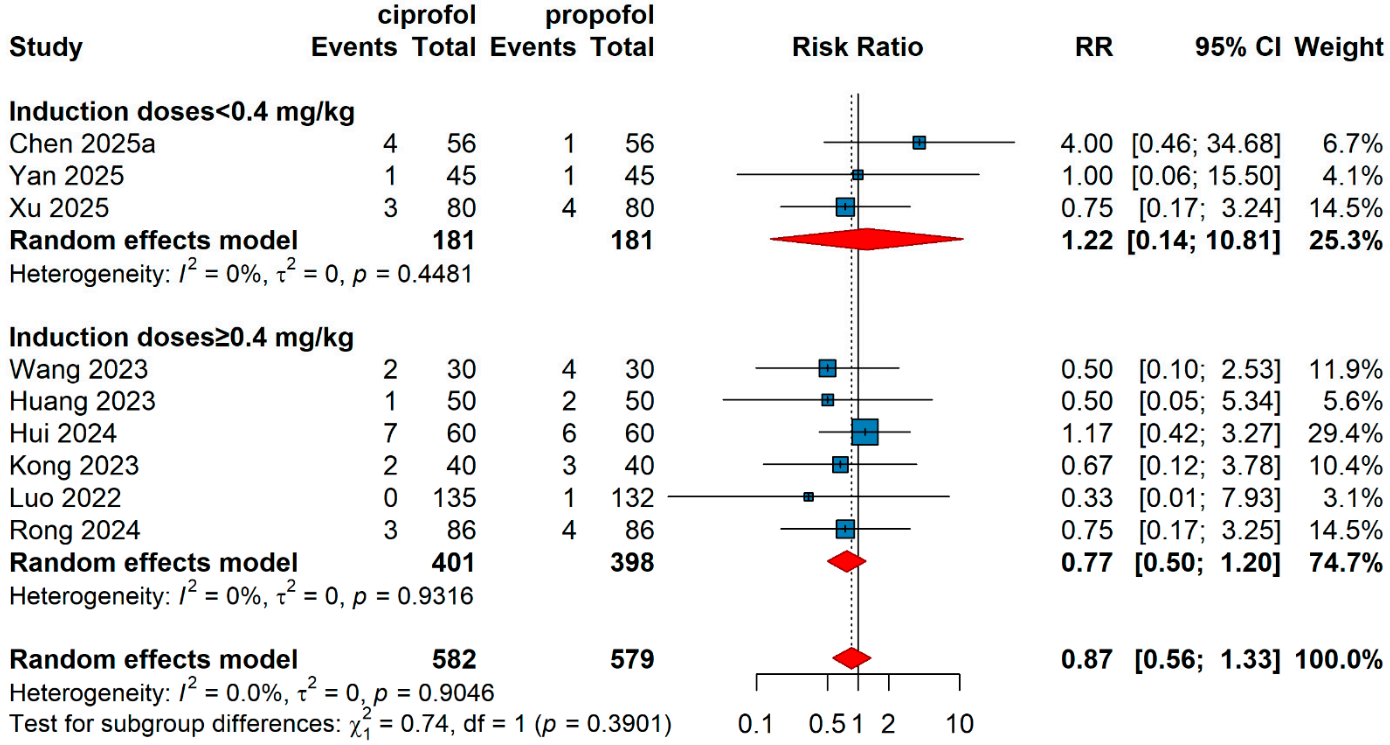

Figure S5. Subgroup analysis of respiratory depression based on ciprofol dose.

Study	ciprofol Events	ciprofol Total	propofol Events	propofol Total	RR	95% CI	Weight
**Induction doses<0.4 mg/kg**							
Wu 2022	4	46	5	46	0.80	[0.23; 2.79]	19.4%
Xu 2025	4	80	24	80	0.17	[0.06; 0.46]	27.7%
**Random effects model**		**126**		**126**	**0.35**	**[0.00; 7313.20]**	**47.1%**
Heterogeneity: $I^2 = 72.6\%$, $\tau^2 = 0.8937$, $p = 0.0559$							
**Induction doses≥0.4 mg/kg**							
Huang 2023	1	50	8	50	0.12	[0.02; 0.96]	7.9%
Wang 2024b	2	30	9	30	0.22	[0.05; 0.94]	14.9%
Ye 2023	1	30	8	30	0.12	[0.02; 0.94]	8.0%
Mei 2024	1	40	8	40	0.12	[0.02; 0.95]	7.9%
Rong 2024	2	86	10	86	0.20	[0.05; 0.89]	14.2%
**Random effects model**		**236**		**236**	**0.17**	**[0.12; 0.24]**	**52.9%**
Heterogeneity: $I^2 = 0\%$, $\tau^2 = 0$, $p = 0.9790$							
**Random effects model**		**362**		**362**	**0.23**	**[0.12; 0.43]**	**100.0%**
Heterogeneity: $I^2 = 0.0\%$, $\tau^2 = 0.0570$, $p = 0.5078$							
Test for subgroup differences: $\chi^2_1 = 0.85$, df = 1 ($p = 0.3556$)							

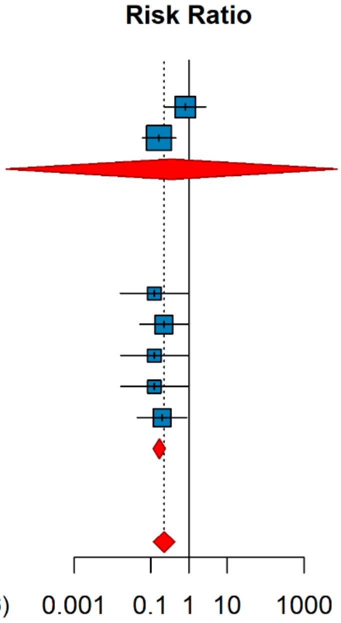

Figure S6. Subgroup analysis of anesthesia induction time based on ciprofol dose.

Study	ciprofol Total	ciprofol Mean	ciprofol SD	propofol Total	propofol Mean	propofol SD	MD	95% CI	Weight
**Induction doses<0.4 mg/kg**									
Wu 2022	46	38.40	16.20	46	37.20	15.60	1.20	[-5.30; 7.70]	7.4%
Li 2025	48	66.00	18.00	48	60.00	42.00	6.00	[-6.93; 18.93]	6.7%
Zhong 2022	46	186.00	50.41	23	150.00	36.00	36.00	[15.30; 56.70]	5.6%
Xu 2025	80	60.00	12.00	80	54.00	18.00	6.00	[1.26; 10.74]	7.6%
**Random effects model**	**220**			**197**			**9.39**	**[-12.33; 31.11]**	**27.3%**
Heterogeneity: $I^2 = 70.2\%$, $\tau^2 = 107.3864$, $p = 0.0180$									
**Induction doses≥0.4 mg/kg**									
Huang 2023	50	51.00	12.60	50	61.80	18.00	-10.80	[-16.89; -4.71]	7.5%
Hui 2024	60	76.23	6.51	60	66.93	6.26	9.30	[7.01; 11.59]	7.7%
Wang 2024b	30	82.29	24.29	30	100.28	37.36	-17.99	[-33.94; -2.04]	6.3%
Ye 2023	30	64.80	17.40	30	51.00	19.20	13.80	[4.53; 23.07]	7.2%
Mei 2024	40	94.80	10.20	40	85.80	13.20	9.00	[3.83; 14.17]	7.5%
Zhang 2025	20	39.85	6.90	20	58.30	6.58	-18.45	[-22.63; -14.27]	7.6%
Nie 2025	76	42.09	4.35	76	22.00	3.02	20.09	[18.90; 21.28]	7.7%
Luo 2022	134	65.53	34.61	133	77.93	87.19	-12.40	[-28.33; 3.53]	6.3%
Rong 2024	86	56.40	16.20	86	54.60	18.00	1.80	[-3.32; 6.92]	7.5%
Zhu 2025	80	72.60	19.80	80	111.60	25.20	-39.00	[-46.02; -31.98]	7.4%
**Random effects model**	**606**			**605**			**-4.08**	**[-17.28; 9.11]**	**72.7%**
Heterogeneity: $I^2 = 98.7\%$, $\tau^2 = 327.6398$, $p < 0.0001$									
**Random effects model**	**826**			**802**			**0.03**	**[-10.57; 10.62]**	**100.0%**
Heterogeneity: $I^2 = 98.2\%$, $\tau^2 = 302.9240$, $p < 0.0001$									
Test for subgroup differences: $\chi^2_1 = 2.25$, df = 1 ($p = 0.1335$)									

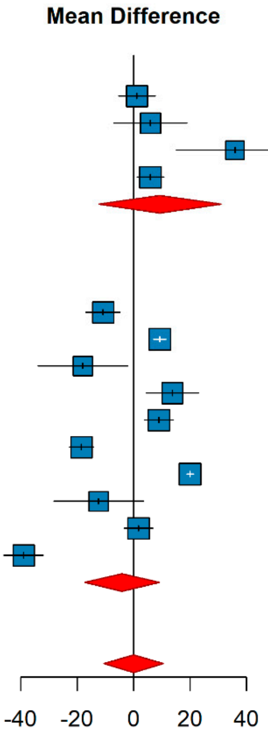

Figure S7. Subgroup analysis of operation time based on ciprofol dose.

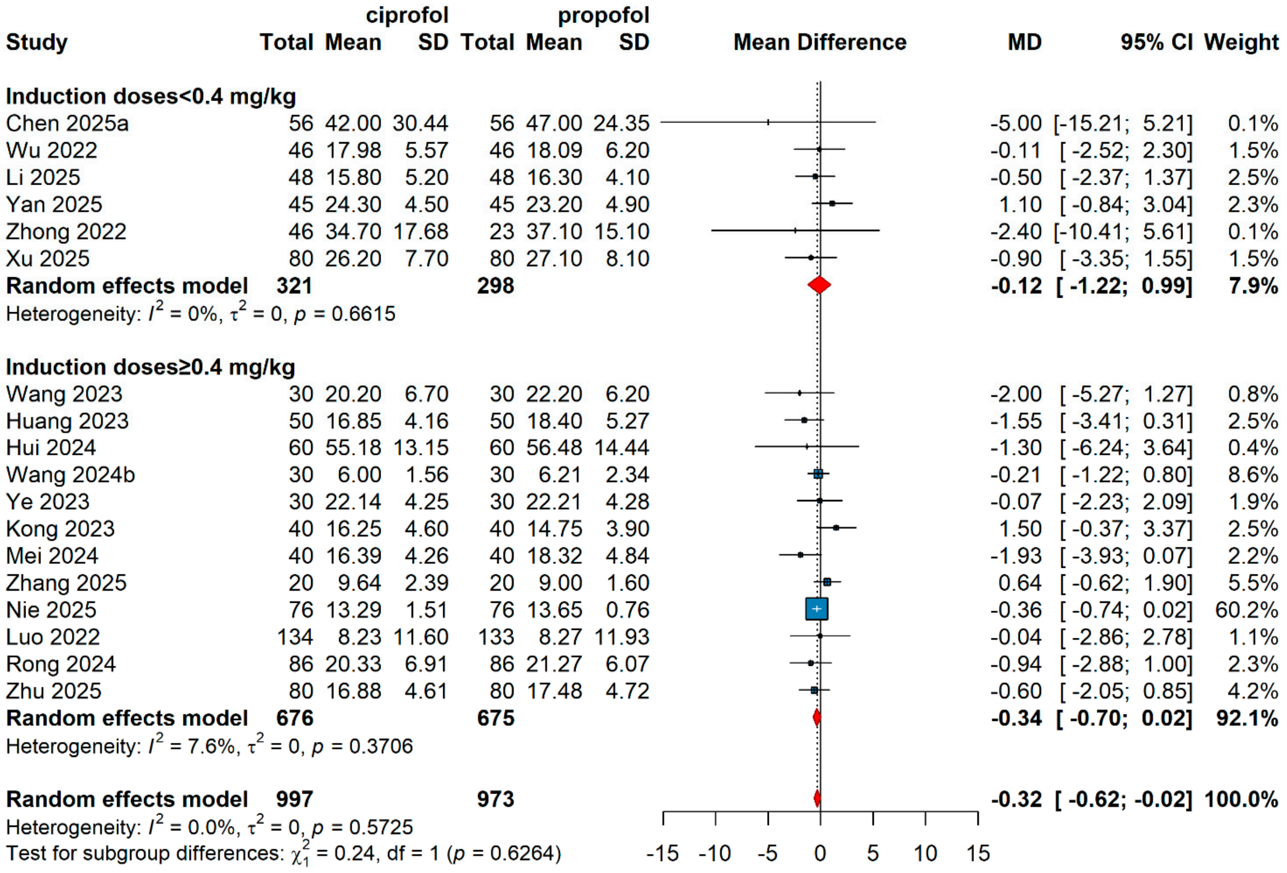

Figure S8. Subgroup analysis of recovery time after surgery based on ciprofol dose.

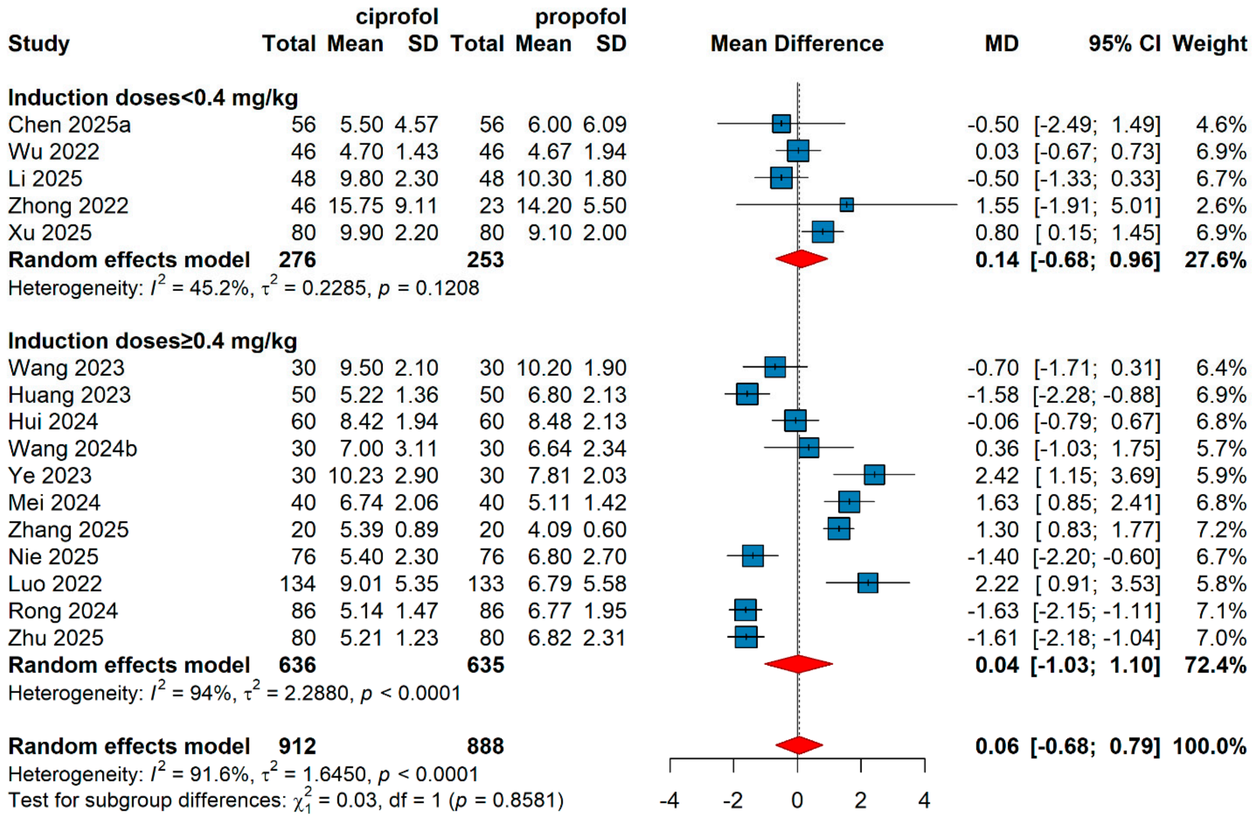

Figure S9. Subgroup analysis of procedural success rate based on ciprofol dose.

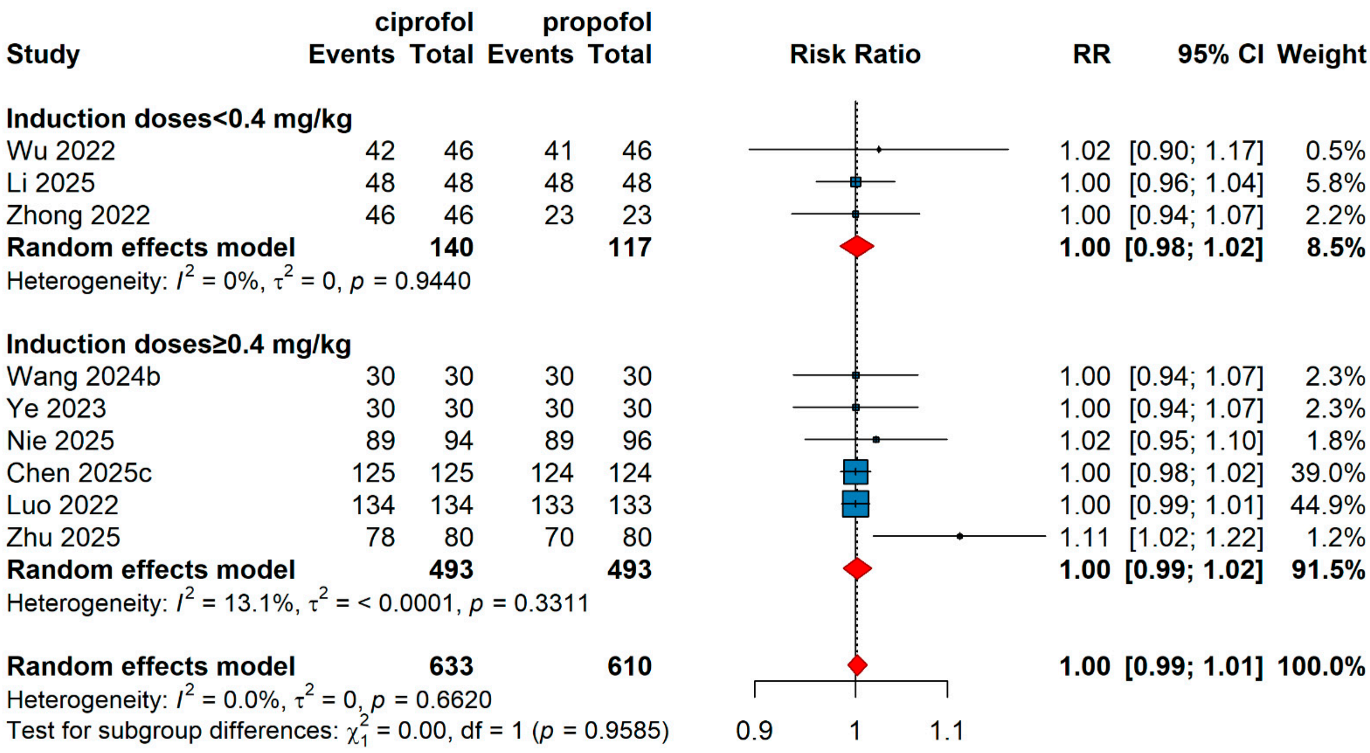

Figure S10. Leave-one-out sensitivity analysis for hypotension outcome.

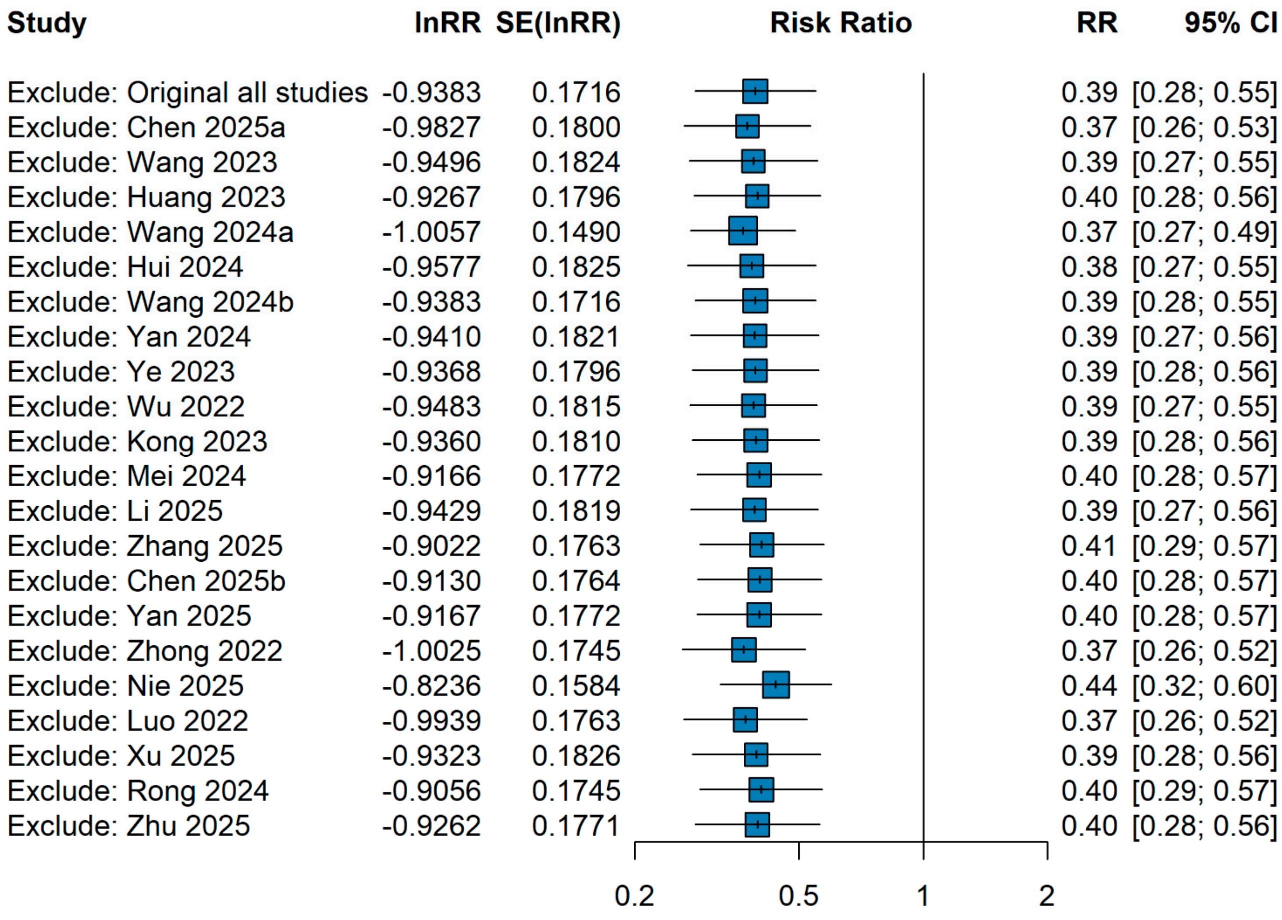

Figure S11. Leave-one-out sensitivity analysis for injection pain outcome.

Study	lnRR	SE(lnRR)	RR	95% CI
Exclude: Original all studies	-1.9916	0.1745	0.14	[0.10; 0.19]
Exclude: Wang 2023	-1.9974	0.1875	0.14	[0.09; 0.20]
Exclude: Huang 2023	-1.9780	0.1795	0.14	[0.10; 0.20]
Exclude: Wang 2024a	-2.0731	0.1727	0.13	[0.09; 0.18]
Exclude: Hui 2024	-1.9920	0.1867	0.14	[0.09; 0.20]
Exclude: Wang 2024b	-1.9830	0.1826	0.14	[0.10; 0.20]
Exclude: Ye 2023	-2.0090	0.1803	0.13	[0.09; 0.19]
Exclude: Wu 2022	-2.0411	0.1899	0.13	[0.09; 0.19]
Exclude: Kong 2023	-2.0498	0.1882	0.13	[0.09; 0.19]
Exclude: Mei 2024	-1.9809	0.1802	0.14	[0.10; 0.20]
Exclude: Li 2025	-2.0783	0.1826	0.13	[0.09; 0.18]
Exclude: Zhang 2025	-1.9546	0.1712	0.14	[0.10; 0.20]
Exclude: Chen 2025b	-1.9733	0.1781	0.14	[0.10; 0.20]
Exclude: Yan 2025	-1.9780	0.1795	0.14	[0.10; 0.20]
Exclude: Zhong 2022	-1.9902	0.1816	0.14	[0.10; 0.20]
Exclude: Nie 2025	-2.0798	0.1891	0.12	[0.09; 0.18]
Exclude: Chen 2025c	-1.9063	0.1577	0.15	[0.11; 0.20]
Exclude: Luo 2022	-1.9907	0.1955	0.14	[0.09; 0.20]
Exclude: Xu 2025	-1.8779	0.1589	0.15	[0.11; 0.21]
Exclude: Rong 2024	-1.9755	0.1788	0.14	[0.10; 0.20]

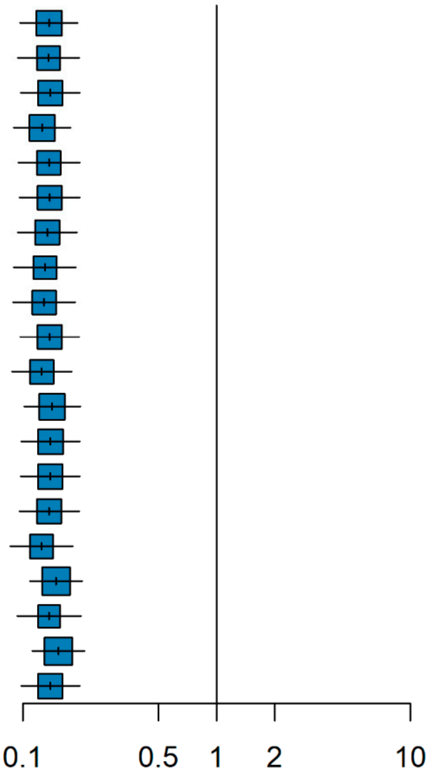

Figure S12. Leave-one-out sensitivity analysis for hypoxemia outcome.

Study	lnRR	SE(lnRR)	RR	95% CI
Exclude: Original all studies	-0.5811	0.2402	0.56	[0.35; 0.90]
Exclude: Chen 2025a	-0.5536	0.2481	0.57	[0.35; 0.93]
Exclude: Huang 2023	-0.5282	0.2397	0.59	[0.37; 0.94]
Exclude: Wang 2024a	-0.6214	0.2599	0.54	[0.32; 0.89]
Exclude: Hui 2024	-0.6811	0.2465	0.51	[0.31; 0.82]
Exclude: Wu 2022	-0.6362	0.2785	0.53	[0.31; 0.91]
Exclude: Kong 2023	-0.5183	0.2798	0.60	[0.34; 1.03]
Exclude: Mei 2024	-0.5757	0.2659	0.56	[0.33; 0.95]
Exclude: Li 2025	-0.5082	0.2586	0.60	[0.36; 1.00]
Exclude: Zhang 2025	-0.5781	0.2609	0.56	[0.34; 0.94]
Exclude: Chen 2025b	-0.5811	0.2402	0.56	[0.35; 0.90]
Exclude: Zhong 2022	-0.5811	0.2402	0.56	[0.35; 0.90]
Exclude: Nie 2025	-0.5102	0.2633	0.60	[0.36; 1.01]
Exclude: Luo 2022	-0.6969	0.1929	0.50	[0.34; 0.73]
Exclude: Zhu 2025	-0.5614	0.2529	0.57	[0.35; 0.94]

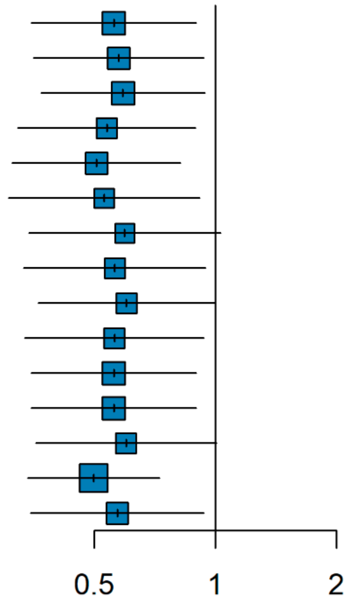

Figure S13. Leave-one-out sensitivity analysis for cough outcome.

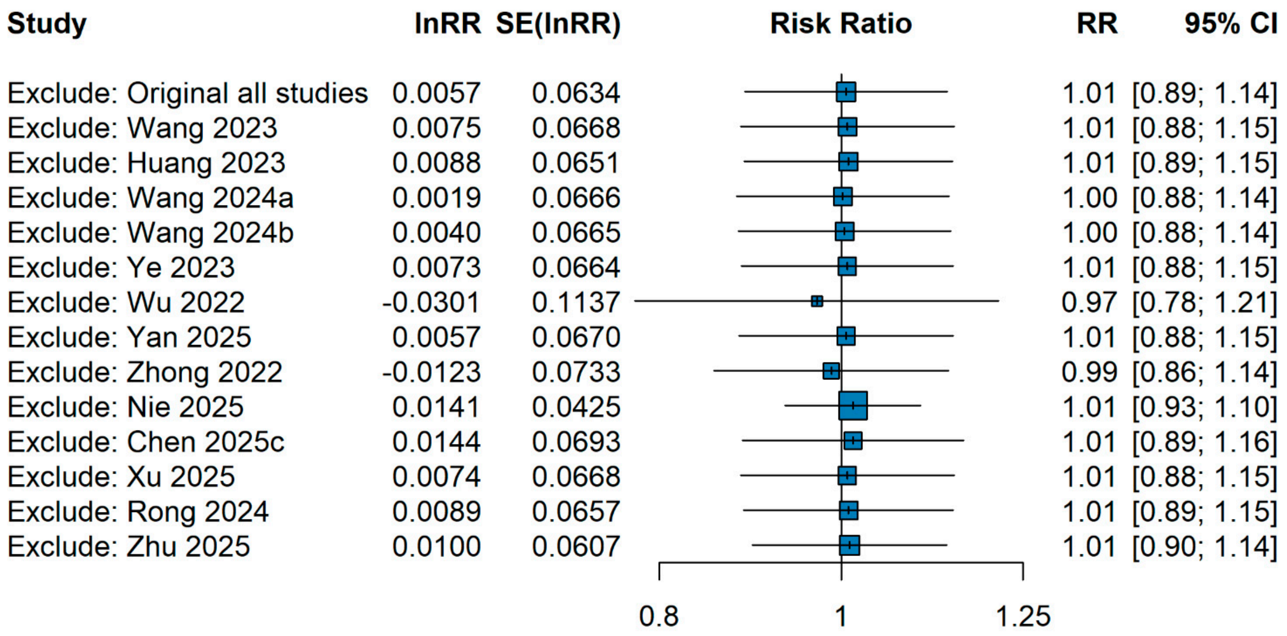

Figure S14. Leave-one-out sensitivity analysis for bradycardia outcome.

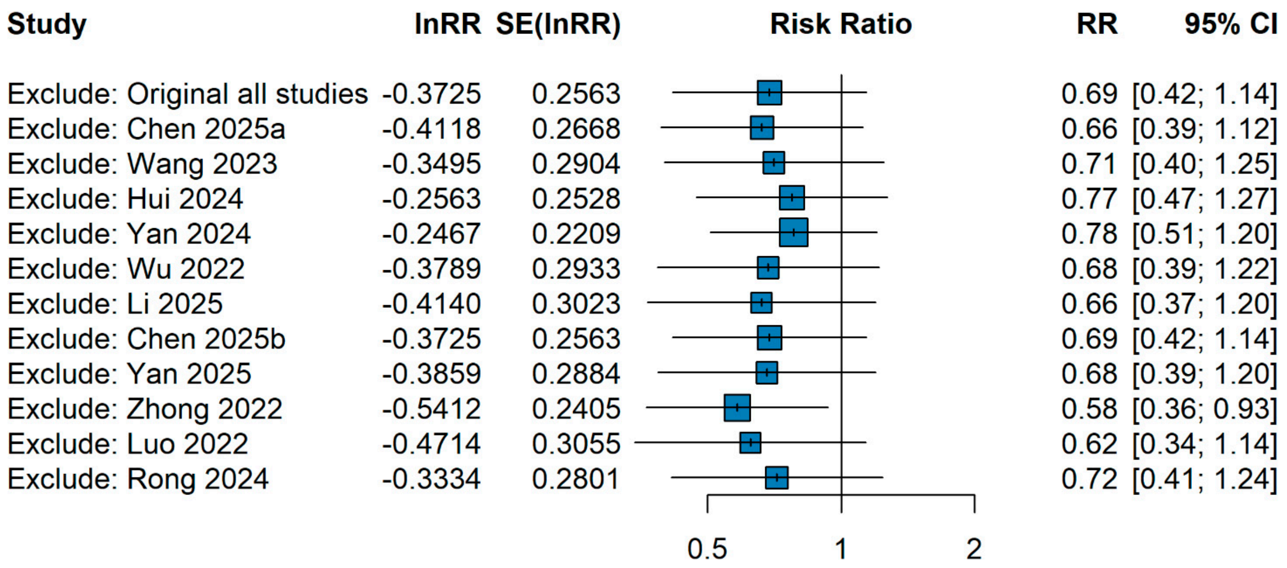

Figure S15. Leave-one-out sensitivity analysis for nausea and vomiting outcome.

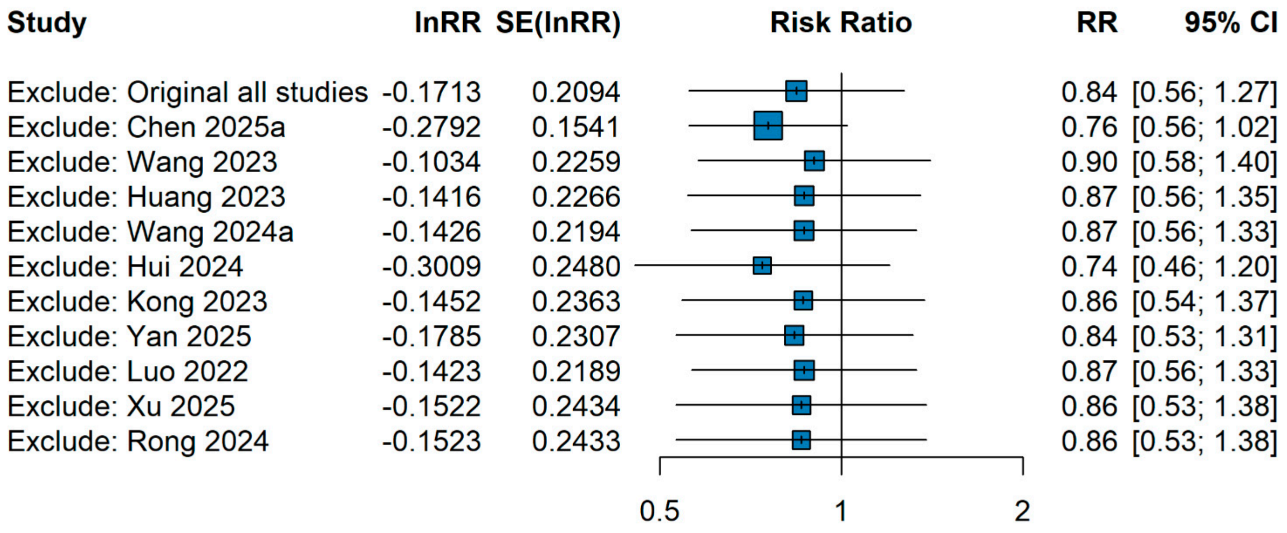

Figure S16. Leave-one-out sensitivity analysis for respiratory depression outcome.

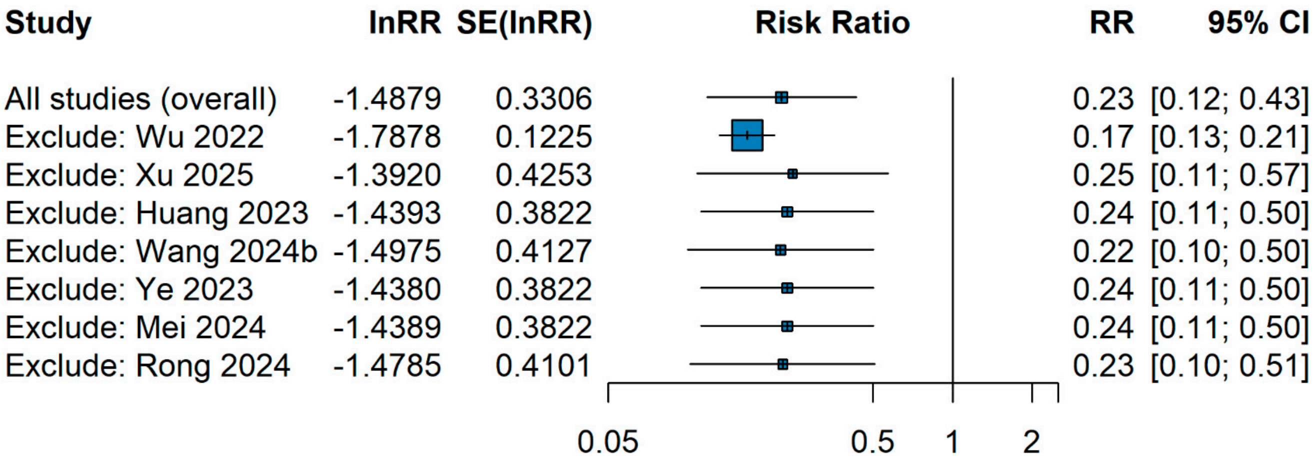

Figure S17. Leave-one-out sensitivity analysis for induction time outcome.

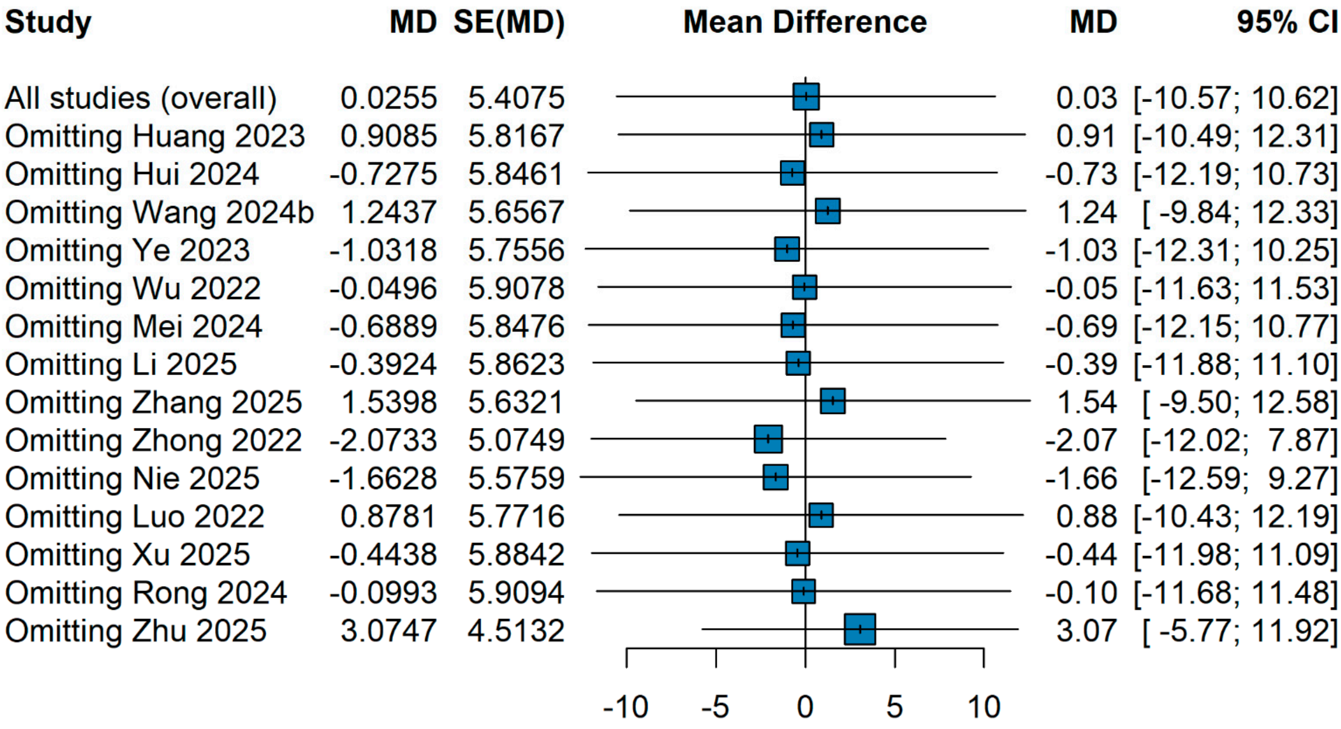

Figure S18. Leave-one-out sensitivity analysis for operation time outcome.

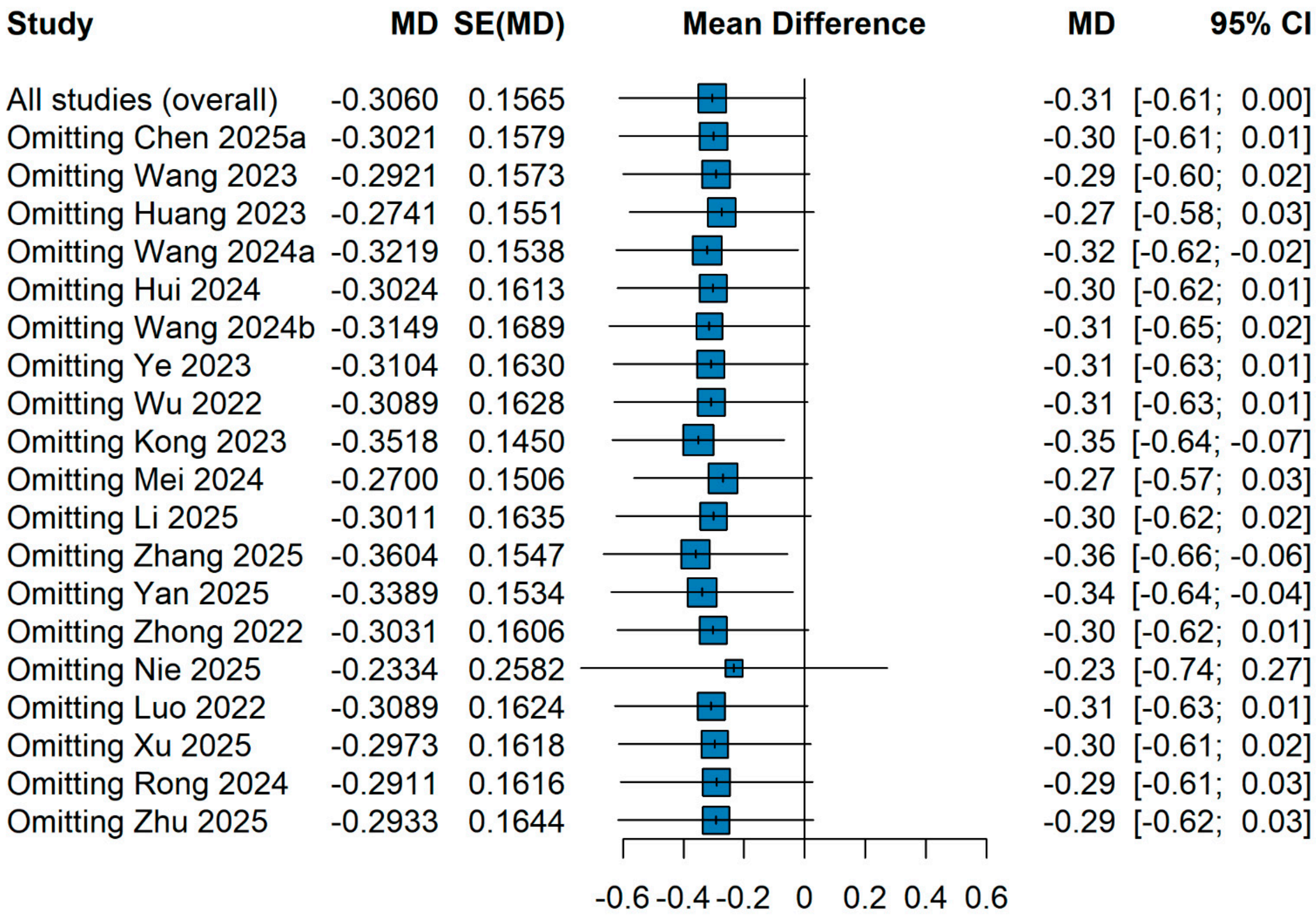

Figure S19. Leave-one-out sensitivity analysis for recovery time outcome.

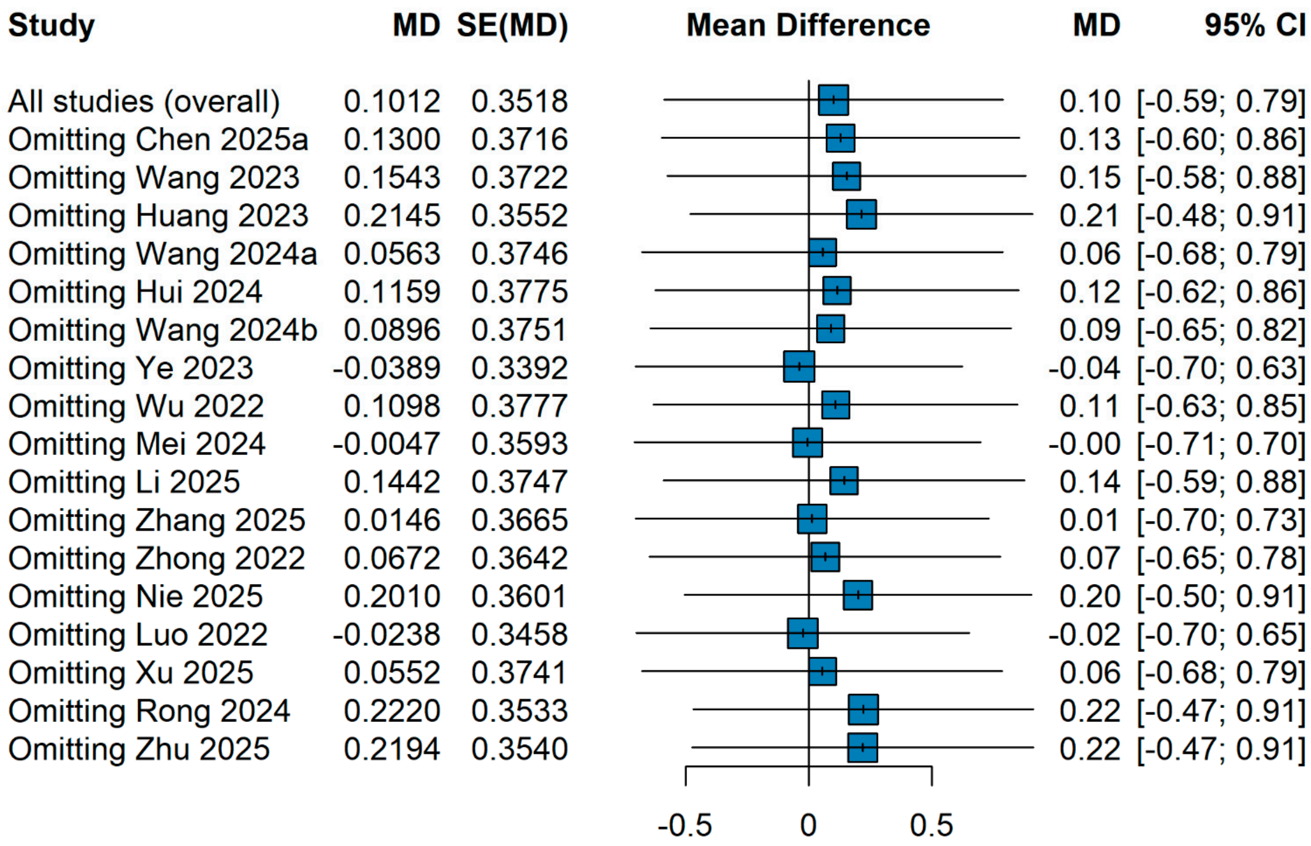

Figure S20. Leave-one-out sensitivity analysis for procedural success rate outcome.

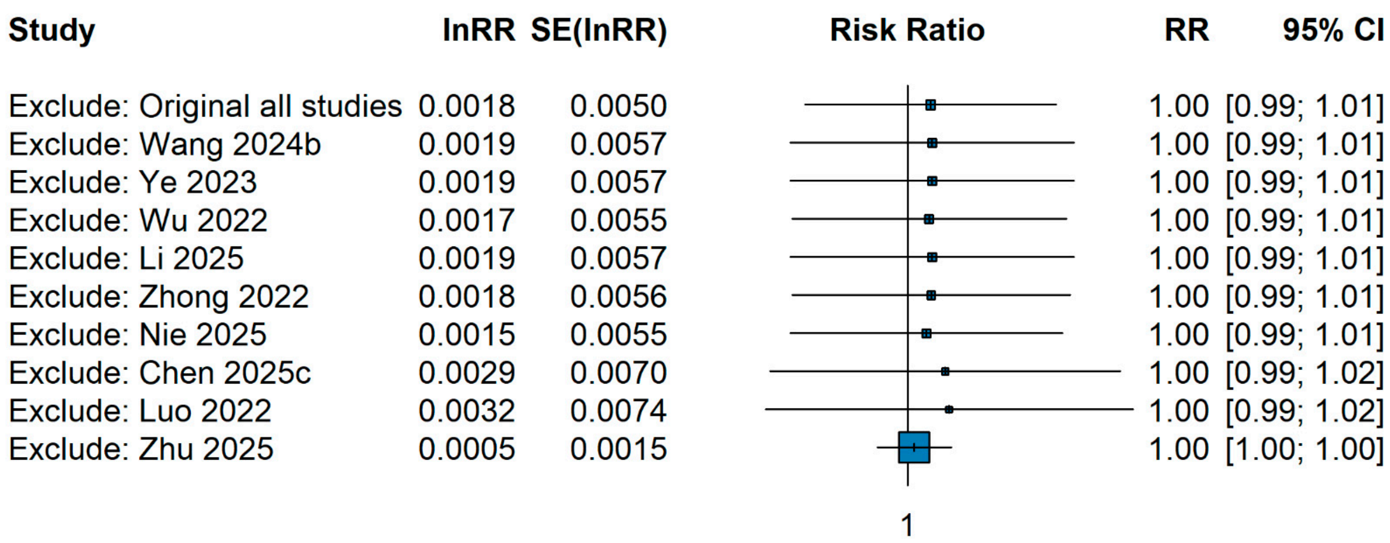

Figure S21. Assessment of publication bias for anesthetic effect via funnel plot and statistical tests. (A) Anesthesia induction time; (B) Operation time; (C) Recovery time.

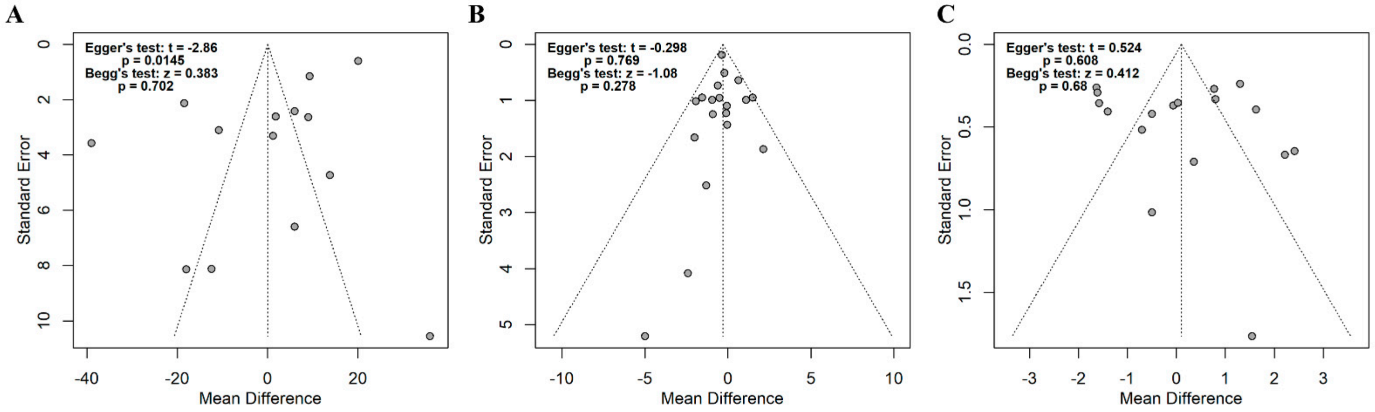

Figure S22. Trim-and-fill analysis of hypotension.

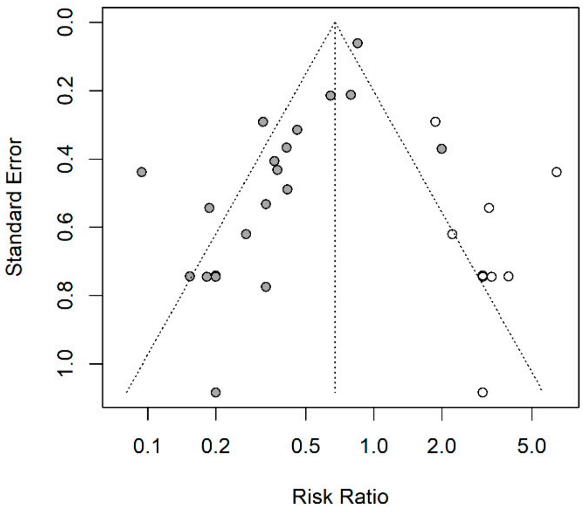


```
Number of studies: k = 30 (with 10 added studies)
Number of observations: o = 3247 (o.e = 1641, o.c = 1606)
Number of events: e = 742

                          RR           95% CI      z p-value
Random effects model 0.6669 [0.4467; 0.9958] -1.98  0.0476

Quantifying heterogeneity (with 95% CIs):
 tau^2 = 0.9326 [0.4933; 2.0925]; tau = 0.9657 [0.7024; 1.4466]
 I^2 = 78.9% [70.4%; 84.9%]; H = 2.18 [1.84; 2.58]

Test of heterogeneity:
      Q d.f.  p-value
 137.25   29 < 0.0001
```

Figure S23. Trim-and-fill analysis of injection pain.

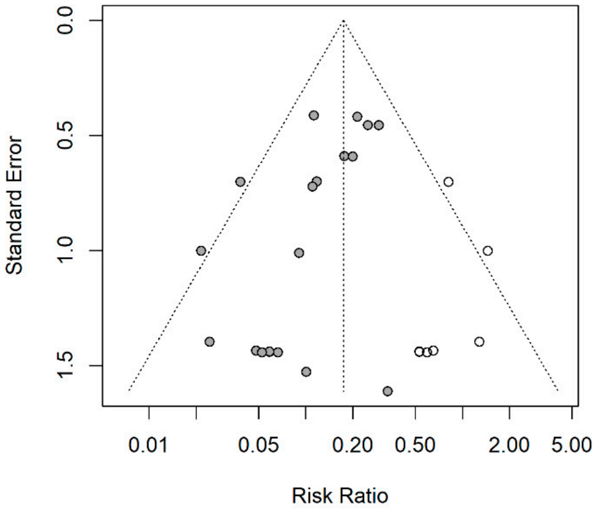


```
Number of studies: k = 26 (with 7 added studies)
Number of observations: o = 3018 (o.e = 1527, o.c = 1491)
Number of events: e = 548

                          RR           95% CI     t  p-value
Random effects model 0.1738 [0.1174; 0.2574] -9.18 < 0.0001

Quantifying heterogeneity (with 95% CIs):
 tau^2 = 0.1642 [0.0000; 1.3636]; tau = 0.4052 [0.0000; 1.1677]
 I^2 = 24.0% [0.0%; 53.2%]; H = 1.15 [1.00; 1.46]

Test of heterogeneity:
     Q d.f. p-value
 32.88   25  0.1340
```

Figure S24. Trim-and-fill analysis of cough.

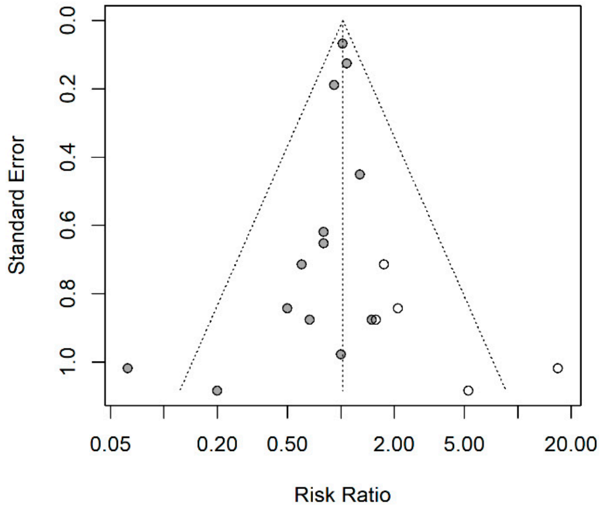


```
Number of studies: k = 18 (with 5 added studies)
Number of observations: o = 2148 (o.e = 1086, o.c = 1062)
Number of events: e = 344

                         RR           95% CI    t p-value
Random effects model 1.0270 [0.8940; 1.1798] 0.41  0.6903

Quantifying heterogeneity (with 95% CIs):
 tau^2 < 0.0001 [0.0000; 2.0659]; tau = 0.0016 [0.0000; 1.4373]
 I^2 = 29.3% [0.0%; 60.0%]; H = 1.19 [1.00; 1.58]

Test of heterogeneity:
     Q d.f. p-value
 24.04   17  0.1184
```

Figure S25. Trim-and-fill analysis of induction time.

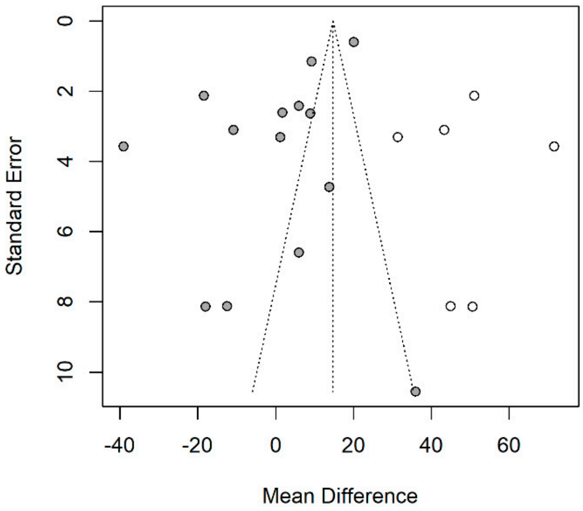


```
Number of studies: k = 20 (with 6 added studies)
Number of observations: o = 2347 (o.e = 1186, o.c = 1161)

                          MD            95% CI    t p-value
Random effects model 14.7533 [1.4596; 28.0470] 2.32  0.0314

Quantifying heterogeneity (with 95% CIs):
 tau^2 = 780.7787 [440.9181; 1696.0754]; tau = 27.9424 [20.9980; 41.1834]
 I^2 = 98.6% [98.4%; 98.9%]; H = 8.58 [7.84; 9.39]

Test of heterogeneity:
       Q d.f.  p-value
 1399.82   19 < 0.0001
```

Reference

1. Chen, X.; Du, W.; Li, Y.; Mu, D.; Ding, T. Effect of Ciprofol on Intraoperative Hypotension in Patients Undergoing Bronchoscopy Procedures. *Chin. J. Anesthesiol.* **2025**, *45*, 415–418. https://doi.org/10.3760/cma.j.cn131073-20240826-00405.
2. Wang, Y.; Chen, L.; Sun, Y.; Chen, L. Comparison of Ciprofol or Propofol Combined with Remifentanil for Painless Fiberoptic Bronchoscopy. *J. Clin. Anesthesiol.* **2023**, *39*, 1050–1054. https://doi.org/10.12089/jca.2023.10.008.
3. Huang, X.; Lin, L. Effect of Ciprofol Combined with Low-Dose Sufentanil for Painless Flexible Bronchoscope. *China Mod. Doctor* **2023**, *61*, 87–90. https://doi.org/10.3969/j.issn.1673-9701.2023.22.021.
4. Wang, L.; Pang, X.; Zheng, Z.; Ding, W.; Wang, Y.; Wang, Y.; Jia, M. Study on the Safety of Anesthesia with Ciprofol Combined with Esketamine in EBUS Examination. *Pract. Pharm. Clin. Rem.* **2024**, *27*, 525–531. https://doi.org/10.14053/j.cnki.ppcr.202407010.
5. Hui, Y.; Tan, S.; Sun, Y. Clinical Trial of Ciprofol Combined with Alfentanil in Patients Undergoing Bronchoscopy. *Chin. J. Clin. Pharmacol.* **2024**, *40*, 2806–2810. https://doi.org/10.13699/j.cnki.1001-6821.2024.19.010.
6. Wang, Y.; Yang, K.; Lai, S. Comparison of the Application of Ciprofol and Propofol Combined with Fentanyl in Painless Bronchoscopy for Conscious Patients in Intensive Care Unit. *China J. Endosc.* **2024**, *30*, 59–65. https://doi.org/10.12235/E20230253.
7. Yan, H.; Jin, L.; Dou, Q.; Liu, X. Application of Ciprofol in Analgesia/Anesthesia with Bronchoscopy Diagnosis and Treatment. *China Med. Pharm.* **2024**, *14*, 165–168. https://doi.org/10.20116/j.issn2095-0616.2024.17.39.
8. Ye, J.; Zhang, J. The Application of Ciprofol Combined with Sufentanil for Deep Sedation in Fiberoptic Bronchoscopy. *J. Wenzhou Med. Univ.* **2023**, *53*, 830–833, 838. https://doi.org/10.3969/j.issn.2095-9400.2023.10.010.
9. Wu, B.; Zhu, W.; Wang, Q.; Ren, C.; Wang, L.; Xie, G. Efficacy and Safety of Ciprofol-Remifentanil versus Propofol-Remifentanil during Fiberoptic Bronchoscopy: A Prospective, Randomized, Double-Blind, Non-Inferiority Trial. *Front. Pharmacol.* **2022**, *13*, 1091579. https://doi.org/10.3389/fphar.2022.1091579.
10. Kong, D.; Song, Y.; Shen, L. Comparison of Ciprofol and Propofol for Fiberoptic Bronchoscopy Performed under General Anesthesia via Laryngeal Mask Airway. *J. Med. Theory Pract.* **2023**, *36*, 4027–4029. https://doi.org/10.19381/j.issn.1001-7585.2023.23.025.
11. Mei, Q.; He, W. Comparative Analysis of Application of Ciprofol Combined with Sufentanil for Painless Fiberoptic Bronchoscopy and Its Effect on HR, MAP and SpO_2. *Smart Healthc.* **2024**, *10*, 45–48. https://doi.org/10.19335/j.cnki.2096-1219.2024.35.012.
12. Li, H.; Xiao, H.; Yang, R.; He, W. Efficacy and Safety Observation of Ciprofol for Painless Fiber Bronchoscopy in Elderly Patients. *J. Pract. Med.* **2025**, *41*, 1217–1223. https://doi.org/10.3969/j.issn.1006-5725.2025.08.019.
13. Zhang, Y.; Yang, L.; Chen, X.; Yu, D.; Zhang, C. Anesthetic Effect of Ciprofol Combined with Remifentanil in Fiberoptic Bronchoscopy for Patients with Pulmonary Tuberculosis. *J. Chengdu Med. Coll.* **2025**, *20*, 256–260. https://doi.org/10.3969/j.issn.1674-2257.2025.02.017.
14. Chen, F.; Wang, L.; Wang, C.; Liu, T.; Chang X.; Liu, W. Clinical Application of Ciprofol Combined with Low-Dose Sufentanil in Painless Bronchoscopy. *J. Cardiovasc. Pulm. Dis.* **2025**, *44*, 506–510. https://doi.org/10.3969/j.issn.1007-5062.2025.05.012.
15. Yan, Y.; Liu, H.; Zheng, M.; Peng, J. Clinical Efficacy Analysis of Combination of Ciprofol and Remifentanil for Painless Fiberoptic Bronchoscopy. *China J. Endosc.* **2025**, *31*, 1–8. https://doi.org/10.12235/E20250085.
16. Zhong, J.; Zhang, J.; Fan, Y.; Zhu, M.; Zhao, X.; Zuo, Z.; Zhou, X.; Miao, C. Efficacy and Safety of Ciprofol for Procedural Sedation and Anesthesia in Non-Operating Room Settings. *J. Clin. Anesth.* **2023**, *85*, 111047. https://doi.org/10.1016/j.jclinane.2022.111047.
17. Nie, J.; Zhang, Y.; Wang, Y.; Fang, L.; Ma, H.; Zhang, Y.; Wang, H. Enhanced Hemodynamic Stability and Patient Satisfaction with Ciprofol-Remifentanil versus Propofol-Remifentanil for Sedation in Shorter-Duration Fiberoptic Bronchoscopy: A Prospective, Randomized, Double-Blind Study. *Front. Med. (Lausanne)* **2025**, *12*, 1498010. https://doi.org/10.3389/fmed.2025.1498010.
18. Chen, F.; Xing, F.; Huang, J.; Zhu, J.; Jiang, Y.; Li, C. Effect of Ciprofol Compared with Propofol on Hemodynamics in Bronchoscope Procedures during Anesthetic Induction: A Randomized Double-Blind Controlled Study. *Front. Med. (Lausanne)* **2025**, *12*, 1545736. https://doi.org/10.3389/fmed.2025.1545736.
19. Luo, Z.; Tu, H.; Zhang, X.; Wang, X.; Ouyang, W.; Wei, X.; Zou, X.; Zhu, Z.; Li, Y.; Shangguan, W. Efficacy and Safety of HSK3486 for Anesthesia/Sedation in Patients Undergoing Fiberoptic Bronchoscopy: A Multicenter, Double-Blind, Propofol-Controlled, Randomized, Phase 3 Study. *CNS Drugs* **2022**, *36*, 301–313. https://doi.org/10.1007/s40263-021-00890-1.
20. Xu, Y.; Wang, Y.; Chen, A.; Wang, L.; Du, J. Effect of Ciprofol Combined with Low-Dose Esketamine in Endobronchial Ultrasound-Guided Transbronchial Needle Aspiration. *J. Clin. Anesthesiol.* **2025**, *41*, 885–887. https://doi.org/10.12089/jca.2025.08.018.
21. Rong, L.; Zhang, Y. Therapeutic Efficacy of Ciprofol Combined with Low-Dose Sufentanil in Bronchoscopic Therapy. *World Latest Med. Inf.* **2024**, *24*, 226–229. https://doi.org/10.3969/j.issn.1671-3141.2024.026.058.
22. Zhu, W.; Chen, Z.; Yang, Z.; Yun, J. A Randomized Parallel-Controlled Study of the Combination of Ciprofol and Low-Dose Sufentanil in Pain-Free Fiberoptic Bronchoscopy. *Guide China Med.* **2025**, *23*, 86–88. https://doi.org/10.15912/j.issn.1671-8194.2025.23.024.
